# The haematological consequences of *Plasmodium vivax* malaria after chloroquine treatment with and without primaquine: a WorldWide Antimalarial Resistance Network systematic review and individual patient data meta-analysis

**DOI:** 10.1186/s12916-019-1386-6

**Published:** 2019-08-01

**Authors:** Robert J. Commons, Julie A. Simpson, Kamala Thriemer, Cindy S. Chu, Nicholas M. Douglas, Tesfay Abreha, Sisay G. Alemu, Arletta Añez, Nicholas M. Anstey, Abraham Aseffa, Ashenafi Assefa, Ghulam R. Awab, J. Kevin Baird, Bridget E. Barber, Isabelle Borghini-Fuhrer, Umberto D’Alessandro, Prabin Dahal, André Daher, Peter J. de Vries, Annette Erhart, Margarete S. M. Gomes, Matthew J. Grigg, Jimee Hwang, Piet A. Kager, Tsige Ketema, Wasif A. Khan, Marcus V. G. Lacerda, Toby Leslie, Benedikt Ley, Kartini Lidia, Wuelton M. Monteiro, Dhelio B. Pereira, Giao T. Phan, Aung P. Phyo, Mark Rowland, Kavitha Saravu, Carol H. Sibley, André M. Siqueira, Kasia Stepniewska, Walter R. J. Taylor, Guy Thwaites, Binh Q. Tran, Tran T. Hien, José Luiz F. Vieira, Sonam Wangchuk, James Watson, Timothy William, Charles J. Woodrow, Francois Nosten, Philippe J. Guerin, Nicholas J. White, Ric N. Price

**Affiliations:** 1 Global Health Division, Menzies School of Health Research and Charles Darwin University, Darwin, Northern Territory Australia; 2 WorldWide Antimalarial Resistance Network (WWARN), Clinical Module, Darwin, Northern Territory Australia; 3 Centre for Epidemiology and Biostatistics, Melbourne School of Population and Global Health, The University of Melbourne, Melbourne, Victoria Australia; 4 Centre for Tropical Medicine and Global Health, Nuffield Department of Clinical Medicine, University of Oxford, Oxford, UK; 5 Shoklo Malaria Research Unit, Mahidol-Oxford Tropical Medicine Research Unit, Faculty of Tropical Medicine, Mahidol University, Mae Sot, Thailand; 6 ICAP, Columbia University Mailman School of Public Health, Addis Ababa, Ethiopia; 7 Addis Ababa University, Addis Ababa, Ethiopia; 8 Armauer Hansen Research Institute, Addis Ababa, Ethiopia; 9 Departamento de Salud Pública, Universidad de Barcelona, Barcelona, Spain; 10 Organización Panamericana de Salud, Oficina de País Bolivia, La Paz, Bolivia; 11 Malaria and Neglected Tropical Diseases Research Team, Bacterial, Parasitic, Zoonotic Diseases Research Directorate, Ethiopian Public Health Institute, Addis Ababa, Ethiopia; 12 Mahidol-Oxford Tropical Medicine Research Unit (MORU), Faculty of Tropical Medicine, Mahidol University, Bangkok, Thailand; 13 Nangarhar Medical Faculty, Nangarhar University, Jalalabad, Afghanistan; 14 Eijkman-Oxford Clinical Research Unit, Jakarta, Indonesia; 15 Infectious Diseases Society Sabah-Menzies School of Health Research Clinical Research Unit, Kota Kinabalu, Sabah Malaysia; 16 Medicines for Malaria Venture, Geneva, Switzerland; 17 Medical Research Council Unit The Gambia at LSTMH, Fajara, The Gambia; 18 WorldWide Antimalarial Resistance Network (WWARN), Oxford, UK; 19 Institute of Drug Technology (Farmanguinhos), Oswaldo Cruz Foundation (FIOCRUZ), Rio de Janeiro, Brazil; 20 Vice-presidency of Research and Reference Laboratories, Oswaldo Cruz Foundation (FIOCRUZ), Rio de Janeiro, Brazil; 21 Liverpool School of Tropical Medicine, Liverpool, UK; 22 Department of Internal Medicine, Tergooi Hospital, Hilversum, the Netherlands; 23 Superintendência de Vigilância em Saúde do Estado do Amapá - SVS/AP, Macapá, Amapá Brazil; 24 Universidade Federal do Amapá – UNIFAP, Macapá, Amapá Brazil; 25 U.S. President’s Malaria Initiative, Malaria Branch, U.S. Centers for Disease Control and Prevention, Atlanta, USA; 26 Global Health Group, University of California San Francisco, San Francisco, USA; 27 Centre for Infection and Immunity Amsterdam (CINEMA), Division of Infectious Diseases, Tropical Medicine and AIDS, Academic Medical Centre, Amsterdam, the Netherlands; 28 Department of Biology, Addis Ababa University, Addis Ababa, Ethiopia; 29 Department of Biology, Jimma University, Jimma, Ethiopia; 30 International Centre for Diarrheal Diseases and Research, Dhaka, Bangladesh; 31 Fundação de Medicina Tropical Dr. Heitor Vieira Dourado, Manaus, Brazil; 32 Universidade do Estado do Amazonas, Manaus, Brazil; 33 Fundação Oswaldo Cruz, Instituto Leônidas e Maria Deane (FIOCRUZ-Amazonas), Manaus, Brazil; 34 Department of Infectious and Tropical Diseases, London School of Hygiene and Tropical Medicine, London, UK; 35 HealthNet-TPO, Kabul, Afghanistan; 36 The Department of Pharmacology and Therapy, Faculty of Medicine, Nusa Cendana University, Kupang, Indonesia; 37 Centro de Pesquisa em Medicina Tropical de Rondônia (CEPEM), Porto Velho, Rondônia Brazil; 38 Universidade Federal de Rondônia (UNIR), Porto Velho, Rondônia Brazil; 39 Division of Infectious Diseases, Tropical Medicine and AIDS, Academic Medical Center, Amsterdam, the Netherlands; 40 Tropical Diseases Clinical Research Center, Cho Ray Hospital, Ho Chi Minh City, Vietnam; 41 Department of Medicine, Kasturba Medical College, Manipal Academy of Higher Education, Madhav Nagar, Manipal, Karnataka India; 42 Manipal McGill Center for Infectious Diseases, Manipal Academy of Higher Education, Manipal, Karnataka India; 43 Department of Genome Sciences, University of Washington, Seattle, USA; 44 Programa de Pós-graduação em Medicina Tropical, Universidade do Estado do Amazonas, Manaus, Brazil; 45 Instituto Nacional de Infectologia Evandro Chagas, Fundação Oswaldo Cruz, Rio de Janeiro, Brazil; 46 Oxford University Clinical Research Unit, Ho Chi Minh City, Vietnam; 47 Federal University of Pará (Universidade Federal do Pará - UFPA), Belém, Pará Brazil; 48 Public Health Laboratory, Department of Public Health, Ministry of Health, Thimphu, Bhutan; 49 Gleneagles Hospital, Kota Kinabalu, Sabah Malaysia

**Keywords:** *Plasmodium vivax*, Chloroquine, Primaquine, Haemoglobin, Pooled analysis, Haemolysis

## Abstract

**Background:**

Malaria causes a reduction in haemoglobin that is compounded by primaquine, particularly in patients with glucose-6-phosphate dehydrogenase (G6PD) deficiency. The aim of this study was to determine the relative contributions to red cell loss of malaria and primaquine in patients with uncomplicated *Plasmodium vivax*.

**Methods:**

A systematic review identified *P. vivax* efficacy studies of chloroquine with or without primaquine published between January 2000 and March 2017. Individual patient data were pooled using standardised methodology, and the haematological response versus time was quantified using a multivariable linear mixed effects model with non-linear terms for time. Mean differences in haemoglobin between treatment groups at day of nadir and day 42 were estimated from this model.

**Results:**

In total, 3421 patients from 29 studies were included: 1692 (49.5%) with normal G6PD status, 1701 (49.7%) with unknown status and 28 (0.8%) deficient or borderline individuals. Of 1975 patients treated with chloroquine alone, the mean haemoglobin fell from 12.22 g/dL [95% CI 11.93, 12.50] on day 0 to a nadir of 11.64 g/dL [11.36, 11.93] on day 2, before rising to 12.88 g/dL [12.60, 13.17] on day 42. In comparison to chloroquine alone, the mean haemoglobin in 1446 patients treated with chloroquine plus primaquine was − 0.13 g/dL [− 0.27, 0.01] lower at day of nadir (*p* = 0.072), but 0.49 g/dL [0.28, 0.69] higher by day 42 (*p* < 0.001). On day 42, patients with recurrent parasitaemia had a mean haemoglobin concentration − 0.72 g/dL [− 0.90, − 0.54] lower than patients without recurrence (*p* < 0.001). Seven days after starting primaquine, G6PD normal patients had a 0.3% (1/389) risk of clinically significant haemolysis (fall in haemoglobin > 25% to < 7 g/dL) and a 1% (4/389) risk of a fall in haemoglobin > 5 g/dL.

**Conclusions:**

Primaquine has the potential to reduce malaria-related anaemia at day 42 and beyond by preventing recurrent parasitaemia. Its widespread implementation will require accurate diagnosis of G6PD deficiency to reduce the risk of drug-induced haemolysis in vulnerable individuals.

**Trial registration:**

This trial was registered with PROSPERO: CRD42016053312. The date of the first registration was 23 December 2016.

**Electronic supplementary material:**

The online version of this article (10.1186/s12916-019-1386-6) contains supplementary material, which is available to authorized users.

## Background

Outside of sub-Saharan Africa, *Plasmodium vivax* is a significant cause of morbidity and mortality in malaria-endemic regions [1–3], resulting in approximately 10 million cases of malaria each year [4]. Anaemia is a common manifestation of vivax malaria, with parasitaemia causing loss of infected and uninfected red blood cells (RBC), as well as reduced RBC production due to dyserythropoiesis [5]. The haematological burden of the disease is compounded by *P*. *vivax*’s ability to form dormant liver stages (hypnozoites) that can reactivate weeks to months after the initial infection, causing multiple relapses [5, 6]. Radical cure of both the erythrocytic and hypnozoite stages of the parasite can prevent recurrent symptomatic *P*. *vivax* infections and thus reduce the cumulative risk of anaemia [7].

Primaquine (PQ), an 8-aminoquinoline compound in use for over 60 years, remains the only widely available drug with activity against hypnozoites, although another 8-aminoquinoline, tafenoquine, was recently licenced by the FDA [8]. 8-Aminoquinolines can cause severe haemolysis in individuals with glucose-6-phosphate dehydrogenase deficiency (G6PDd), an inherited enzymopathy caused by genetic polymorphisms in the X chromosome. The risk of drug-induced haemolysis relates to the dose of PQ and an individual’s genetic polymorphism [9–11]. In general, routine testing for G6PDd is unavailable in most endemic areas and concerns regarding severe haemolysis are a major barrier to widespread clinical use of PQ [12, 13].

The relative contributions of malaria itself and PQ treatment to haemoglobin reductions in patients with vivax malaria are poorly defined. This study aimed to determine the degree of haemoglobin reduction following chloroquine (CQ), the standard blood schizontocidal treatment of vivax malaria [14] and to quantify any additional reduction relating to haemolysis from PQ co-administration.

## Methods

### Search strategy and selection criteria

A systematic search was undertaken in MEDLINE, Web of Science, Embase and the Cochrane Database of Systematic Reviews according to the Preferred Reporting Items for Systematic Reviews and Meta-Analyses (PRISMA) guidelines (Additional file 1: Checklist S1). Prospective therapeutic efficacy trials of treatment of uncomplicated vivax malaria with a minimum of 28 days follow-up, published between 1 January 2000 and 22 March 2017, in any language were identified (Additional file 1: Box S1) [15]. Investigators of eligible studies were invited to participate in an individual patient data meta-analysis and contribute data from similar unpublished studies.

Studies were included in the analysis if they enrolled patients with *P. vivax* monoinfection treated with CQ, alone or with PQ, and recorded haemoglobin (Hb) or haematocrit at baseline. Studies of pregnant women and treatment with adjunctive antimalarials were excluded. Individual patient data were shared on the WorldWide Antimalarial Resistance Network (WWARN) repository, anonymised and standardised [16]. The review protocol was registered in the International Prospective Register of Systematic Reviews (PROSPERO: CRD42016053312).

### Procedures

The doses of CQ and PQ were calculated from the number of tablets given to each patient, or the study protocol if tablet numbers were unavailable. Patient records were excluded if CQ was not administered; PQ was administered after day 0; no Hb or haematocrit was recorded on day 0; adjunctive antimalarials were administered; *P. vivax* was not present at day 0; information on the dose given, parasitaemia, age or gender was unavailable; the CQ treatment course was incomplete; mixed infections were present at day 0; or PQ was dosed intermittently.

G6PD status was recorded when reported, and deficiency was diagnosed by either a qualitative assay (fluorescent spot test or the CareStart® rapid diagnostic test) or a quantitative assay (spectrophotometry). G6PDd was defined as an enzyme activity less than 30% (Additional file 1: Table S1).

Study sites were categorised into regions of long or short *P*. *vivax* relapse periodicity [17], with regions of short relapse periodicity considered to have a median time to relapse of ≤ 47 days. To avoid confounding from early treatment failure, recurrence was defined as vivax parasitaemia between days 7 and 42. Daily PQ mg/kg dose was defined as low dose if < 0.5 mg/kg/day and high dose if ≥ 0.5 mg/kg/day.

When only the haematocrit was available, it was converted to Hb according to the equation [18]:

$$ \mathrm{Hb}\ \left(\mathrm{g}/\mathrm{dL}\right)=\left(\mathrm{haematocrit}\ \left(\%\right)-5.62\right)/2.6 $$

Where multiple Hb measurements were recorded on a single day, the minimum value was used.

### Statistical analysis

Linear mixed effects modelling of the Hb versus time profiles (described below) was used to derive the primary endpoint of the mean drop in Hb from day 0 (baseline) to the day of the nadir and the secondary endpoints of the mean change in Hb from baseline to day 7 and day 42. In addition, two safety outcomes identified patients at risk of poor clinical outcome: a Hb fall of > 25% from a baseline of ≥ 7 g/dL to a Hb < 7 g/dL (defined as a clinically significant fall) and an absolute fall in Hb of > 5 g/dL. The safety outcomes were assessed at day 2 or 3 (day 2/3), day 7 ± 2 days (day 7) and day 28 ± 3 days (day 28).

Statistical analyses were done using Stata v15 (StataCorp) and R version 3.4.0 (R Foundation for Statistical Computing), according to an a priori statistical analysis plan [19]. The mean Hb-time response following treatment was estimated using a linear mixed effects model [20] with non-linear terms, derived by fractional polynomial regression [21, 22]; with fixed effects for age, gender, baseline parasitaemia, total CQ dose (mg/kg), relapse periodicity and PQ use; and with random effects fitted to the terms for time according to an individual within each study site. The interaction between PQ use and time was included in order to capture the different time course of Hb responses following the two regimens CQ or CQ+PQ. In the subgroup of patients treated with PQ, the effect of the daily mg/kg PQ dose on Hb response was estimated using a similar linear mixed effects model. The primary analysis was repeated in subgroups of patients with documented normal G6PD status and unknown G6PD status and by gender. Additional factors associated with the change in haemoglobin between day 0 and day of nadir were assessed using a linear regression model with shared frailty for the study site.

A sensitivity analysis was undertaken to assess potential selection bias, removing one study at a time and calculating the coefficient of variation in the estimates of the primary analysis. Baseline characteristics of included studies were also compared to studies that were targeted but not available for inclusion.

The effect of delayed parasite clearance (defined as persistence of parasitaemia until day 2 or later) on Hb at day of nadir and day 42 and the effect of recurrence between days 7 and 42 on Hb at day 42 were assessed using separate linear mixed effects models similar to the model above with the interaction between PQ and time replaced by interactions between delayed parasite clearance or recurrence and time. In the model of recurrence between days 7 and 42, patients with early treatment failure, late clinical failure prior to day 7 or persistent parasitaemia between days 4 and 6 were excluded from the analysis.

A descriptive table of safety outcomes was presented to provide commonly reported parameters of the Hb response in published clinical trials; the numbers of patients available for these summary statistics varied according to the time point presented. There were insufficient numbers of patients experiencing either of the safety outcomes to conduct multivariable analyses of the haemolytic risk attributable to PQ.

## Results

**Fig. 1:**
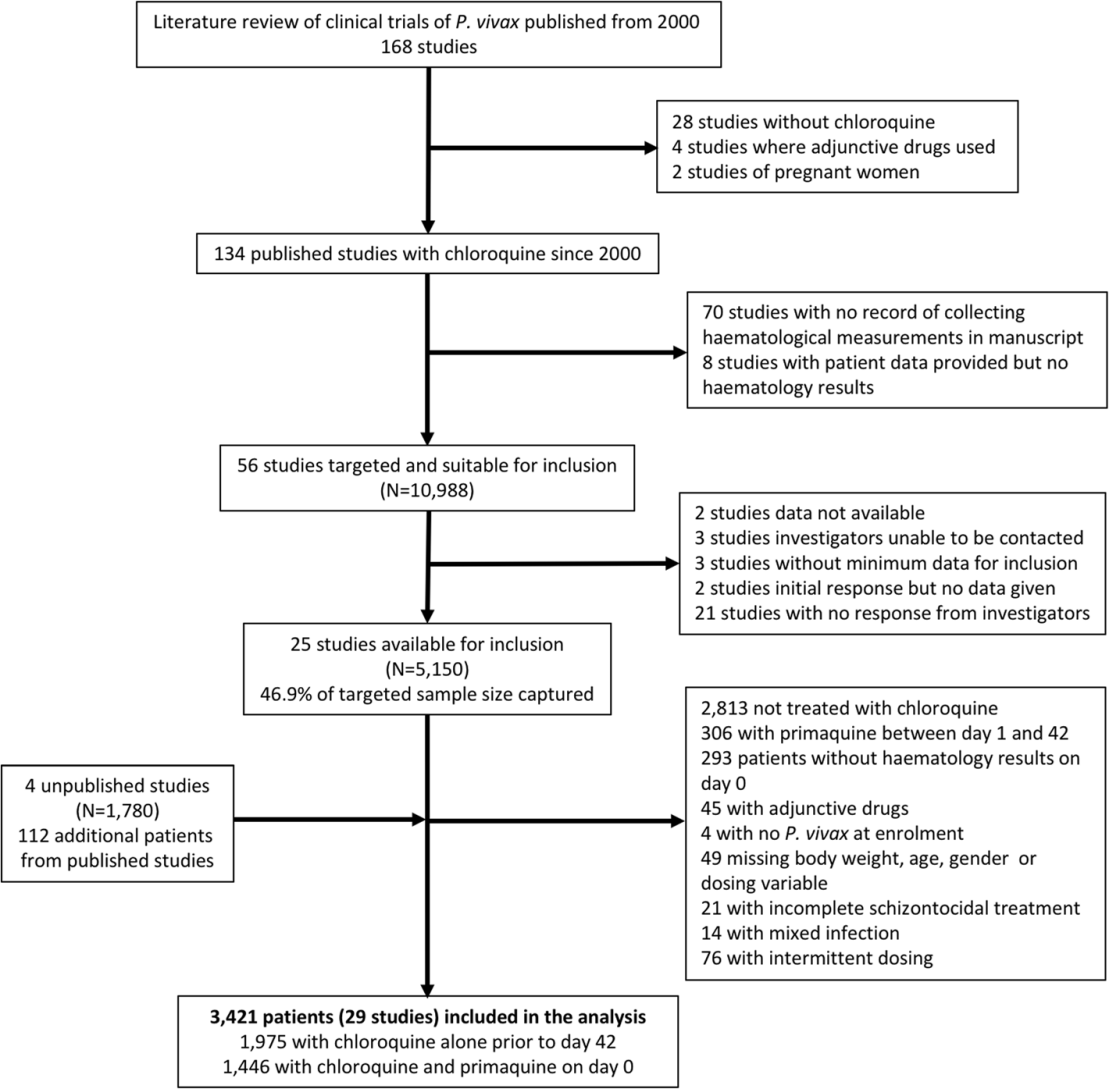
Study flowchart

Between 1 January 2000 and 22 March 2017, there were 168 published *P. vivax* clinical trials of which 134 (79.8%) included patients treated with CQ and 56 (33.3%) provided information on Hb concentration or haematocrit. Individual patient data were available for 5150 (46.9%) patients from 25 of these studies plus 1892 additional patients (1780 from four unpublished studies and 112 from published studies). Of the 7042 patients with available data, 2813 (39.9%) were not treated with CQ, 306 (4.3%) were treated with PQ after day 0 and 502 (7.1%) were excluded for other reasons (Fig. 1 and Additional file 1: Table S1–S3). Of the remaining 3421 patients, 1975 (57.7%) were treated with CQ alone and 1446 (42.3%) with CQ+PQ [23–51].

Patients were followed for 28 days in 14 studies (*n* = 1841), 29 to 42 days in seven studies (*n* = 388) and more than 42 days in eight studies (*n* = 1192). In total, G6PD status was normal in 1692 (49.5%) patients, deficient or borderline deficient in 28 (0.8%) and unknown in 1701 (49.7%) (Additional file 1: Table S4). All G6PD-deficient and borderline patients were identified prior to treatment and were administered CQ alone, except for one deficient patient who was treated with CQ+PQ and was diagnosed post hoc. Target PQ regimens are described in Additional file 1: Table S5.

The majority of patients were male (64.6%, 2211/3421). The median age of patients was 19 years (inter-quartile range (IQR) 9–32), with 1314 (38.4%) patients younger than 15 years (Table 1). Most of the patients were enrolled from the Asia-Pacific region (2247, 65.7%), with 598 (17.5%) enrolled from The Americas and 576 (16.8%) from the Horn of Africa (Additional file 1: Figure S1). Compared to patients treated with CQ, those treated with CQ+PQ tended to be older, have lower baseline parasitaemias and be more likely to come from areas of short relapse periodicity (Table 1). Baseline characteristics of G6PD normal patients and patients with unknown G6PD status are described separately in Additional file 1: Table S6–S7. Compared to the studies that were targeted but not included, included studies were conducted more recently, enrolled younger populations and included more equal proportions of male and female patients (Additional file 1: Table S8).

**Table 1:** Demographics, baseline characteristics and baseline haemoglobin measurements

	Chloroquine alone			Chloroquine plus primaquine			Overall		
	Number (%)*	Mean Hb (SD)	Range	Number (%)*	Mean Hb (SD)	Range	Number (%)*	Mean Hb (SD)	Range
Overall	1975 (100)	12.2 (2.1)	6.0 to 18.7	1446 (100)	12.7 (2.1)	4.0 to 19.0	3421 (100)	12.4 (2.1)	4.0 to 19.0
Parasitaemia, parasites per uL; median (IQR)	3400 (1261, 8290)			2700 (912, 7040)			3104 (1137, 8000)		
Gender									
Female	772 (39.1)	11.8 (1.9)	6.0 to 17.4	438 (30.3)	11.7 (1.8)	4.0 to 17.4	1210 (35.4)	11.7 (1.9)	4.0 to 17.4
Male	1203 (60.9)	12.5 (2.1)	6.6 to 18.7	1008 (69.7)	13.1 (2.1)	4.9 to 19.0	2211 (64.6)	12.8 (2.1)	4.9 to 19.0
Age category, years									
< 5	225 (11.4)	10.7 (2.0)	6.0 to 16.6	72 (5.0)	10.3 (1.8)	4.9 to 14.1	297 (8.7)	10.6 (2.0)	4.9 to 16.6
5 to < 15	691 (35.0)	11.6 (1.8)	6.6 to 17.4	326 (22.5)	11.5 (1.6)	5.5 to 16.3	1017 (29.7)	11.6 (1.8)	5.5 to 17.4
≥ 15	1059 (53.6)	13.0 (1.9)	6.2 to 18.7	1048 (72.5)	13.2 (2.0)	4.0 to 19.0	2107 (61.6)	13.1 (2.0)	4.0 to 19.0
Weight category, kg									
5 to < 15	195 (9.9)	10.4 (1.9)	6.0 to 16.3	83 (5.7)	10.3 (1.6)	5.2 to 13.4	278 (8.1)	10.4 (1.8)	5.2 to 16.3
15 to < 25	440 (22.3)	11.5 (1.9)	6.9 to 16.6	172 (11.9)	11.1 (1.6)	4.9 to 15.9	612 (17.9)	11.4 (1.8)	4.9 to 16.6
25 to < 35	182 (9.2)	11.7 (1.6)	6.6 to 16.2	94 (6.5)	11.7 (1.6)	7.5 to 15.1	276 (8.1)	11.7 (1.6)	6.6 to 16.2
35 to < 45	196 (9.9)	12.1 (1.9)	6.5 to 17.4	153 (10.6)	12.1 (1.9)	5.8 to 17.1	349 (10.2)	12.1 (1.9)	5.8 to 17.4
45 to < 55	404 (20.5)	12.9 (1.9)	6.2 to 18.7	338 (23.4)	12.9 (1.9)	5.4 to 18.1	742 (21.7)	12.9 (1.9)	5.4 to 18.7
55 to < 80	484 (24.5)	13.1 (1.9)	7.0 to 18.1	508 (35.1)	13.6 (1.9)	4.0 to 19.0	992 (29.0)	13.3 (1.9)	4.0 to 19.0
≥ 80	74 (3.7)	13.8 (1.3)	9.9 to 16.5	98 (6.8)	14.0 (1.7)	8.2 to 17.9	172 (5.0)	13.9 (1.5)	8.2 to 17.9
G6PD status									
Normal	856 (43.3)	12.4 (1.9)	6.5 to 18.1	836 (57.8)	12.8 (2.0)	5.4 to 19.0	1692 (49.5)	12.6 (2.0)	5.4 to 19.0
Borderline	3 (0.2)	13.9 (1.1)	13.1 to 15.2	0 (0)	–	–	3 (0.1)	13.9 (1.1)	13.1 to 15.2
Deficient	24 (1.2)	12.4 (1.8)	8.6 to 15.7	1 (0.1)	14.0 (−)	14.0 to 14.0	25 (0.7)	12.4 (1.8)	8.6 to 15.7
Not known	1092 (55.3)	12.1 (2.2)	6.0 to 18.7	609 (42.1)	12.5 (2.2)	4.0 to 18.9	1701 (49.7)	12.2 (2.2)	4.0 to 18.9
Relapse periodicity									
Long	1360 (68.9)	12.3 (2.1)	6.0 to 18.1	627 (43.4)	13.4 (1.9)	4.0 to 18.9	1987 (58.1)	12.6 (2.1)	4.0 to 18.9
Short	615 (31.1)	12.1 (2.0)	6.2 to 18.7	819 (56.6)	12.2 (2.1)	4.9 to 19.0	1434 (41.9)	12.2 (2.0)	4.9 to 19.0
Geographical region									
Asia-Pacific	1114 (56.4)	11.9 (1.9)	6.2 to 18.7	1133 (78.4)	12.5 (2.1)	4.9 to 19.0	2247 (65.7)	12.2 (2.0)	4.9 to 19.0
The Americas	285 (14.4)	12.5 (2.0)	7.0 to 17.4	313 (21.6)	13.5 (1.8)	4.0 to 18.9	598 (17.5)	13.0 (2.0)	4.0 to 18.9
Africa	576 (29.2)	12.7 (2.2)	6.0 to 18.1	0 (0)	–	–	576 (16.8)	12.7 (2.2)	6.0 to 18.1

*Hb* haemoglobin, *SD* standard deviation, *IQR* inter-quartile range

*Number of patients (percentage of total patients in group) unless otherwise specified

### Baseline haemoglobin

The mean Hb at baseline was 12.2 g/dL (SD 2.1) in patients receiving CQ and 12.7 g/dL (SD 2.1) in patients receiving CQ+PQ. Overall, 11.3% (385/3421) of patients were anaemic at baseline (Hb < 10 g/dL), including 13.1% (259/1975) in those subsequently treated with CQ and 8.7% (126/1446) in those treated with CQ+PQ. Severe anaemia (Hb < 7 g/dL) was present in 0.8% (26/3421) of patients. The odds of anaemia at baseline was greater in females (adjusted odds ratio (AOR) = 1.34 [95% CI 1.05, 1.71]) and patients who were younger than 5 years (AOR = 10.37 [6.09, 17.67]), G6PD deficient (AOR = 2.88 [1.14, 7.32]) and enrolled in regions of short relapse periodicity (AOR = 1.94 [1.01, 3.71]) (Additional file 1: Table S9).

### Haemoglobin-time profile

The Hb profile between baseline and day 42 was modelled from 9684 Hb measurements in 1975 patients treated with CQ alone and 6029 Hb measurements in 1446 patients treated with CQ+PQ. Patients treated with CQ alone had a median [IQR] of 7 [5-9] Hb measurements, and patients treated with CQ+PQ had a median [IQR] of 9 [3-10] Hb measurements.

### Haemoglobin profile following treatment with chloroquine alone

**Fig. 2:**
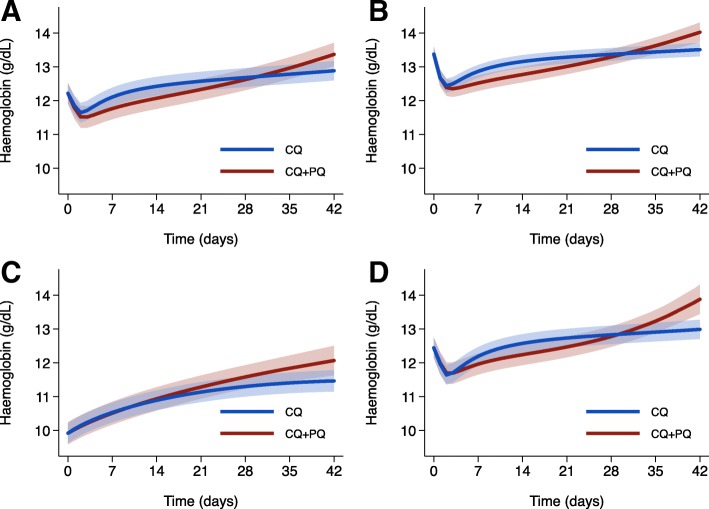
Mean haemoglobin-time profiles for **a** any baseline haemoglobin, **b** baseline ≥ 11.5 g/dL, **c** baseline < 11.5 g/dL and **d** normal G6PD status. Figures derived from the linear mixed effects model with fractional polynomial terms for time. Profiles for chloroquine (CQ) alone and chloroquine plus primaquine (CQ+PQ) adjusted to the same baseline haemoglobin. Shaded regions show 95% confidence intervals. In total, 1975 patients were treated with CQ alone and 1446 with CQ+PQ; in patients with baseline Hb ≥ 11.5 g/dL, the corresponding figures were 1277 and 1063; in patients with baseline Hb < 11.5 g/dL, the corresponding figures were 698 and 383; and in patients with normal G6PD status, the corresponding figures were 856 and 836

In patients treated with CQ alone, the mean Hb fell from baseline to a nadir on day 2, with a fall of 0.58 g/dL from a mean of 12.22 g/dL [95% CI 11.93, 12.50] to 11.64 g/dL [11.36, 11.93] (Fig. 2). Following the nadir, the Hb rose thereafter. By day 42, the mean Hb was 12.88 g/dL [12.60, 13.17], 0.67 g/dL above baseline.

**Fig. 3:**
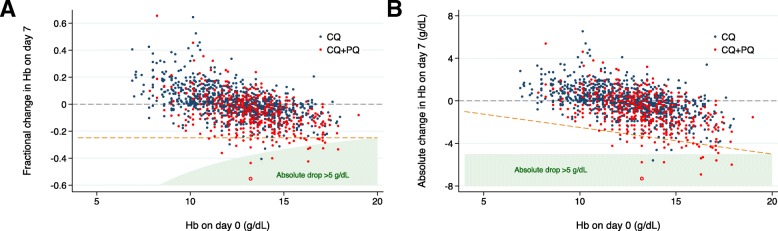
Relationship between haemoglobin at baseline and day 7 as **a** fractional change and **b** absolute change. One thousand two hundred twenty-two patients were treated with chloroquine (CQ) alone and 539 with chloroquine plus primaquine (CQ+PQ). The open circle represents the single patient with a clinically significant fall > 25% to < 7 g/dL at day 7 (female patient with normal G6PD status). The dashed orange line represents a fractional fall of 25%. The baseline Hb correlated negatively with the fractional change in Hb at day 7 (*r* = − 0.521 [95% CI − 0.554 to − 0.486], *p* < 0.0001)

**Fig. 4:**
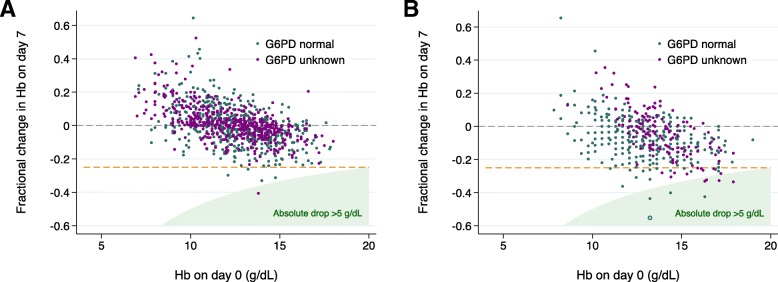
Fractional change in haemoglobin between baseline and day 7 following **a** chloroquine and **b** chloroquine plus primaquine. In patients treated with chloroquine alone, 608 had normal G6PD status and 588 had unknown G6PD status. In patients treated with chloroquine plus primaquine, 389 had normal G6PD status and 150 had unknown G6PD status. The open circle represents the single patient with a clinically significant fall > 25% to < 7 g/dL at day 7 (female patient with normal G6PD status). The dashed orange line represents a fractional fall of 25%

The magnitude and direction of the change in Hb from baseline to day 2 or day 7 varied with the baseline Hb, with a high baseline Hb correlated with a large fall in Hb (Figs. 3 and 4 and Additional file 1: Figure S2). Only 32.8% (136/415) of patients with a baseline Hb < 11.5 g/dL fell below their baseline Hb during the first 7 days compared with 70.9% (565/797) of those with a baseline Hb ≥ 11.5 g/dL (Fig. 2).

### Haemoglobin profile following treatment with chloroquine and primaquine

The nadir Hb in patients treated with CQ+PQ occurred on day 3, with the mean Hb rising throughout the subsequent follow-up (Fig. 2).

Compared to patients treated with CQ alone, those treated with CQ+PQ had a lower Hb at nadir (mean difference − 0.13 g/dL [95% CI − 0.27, 0.01], *p* = 0.072) and day 7 (− 0.34 g/dL [− 0.46, − 0.23], *p* < 0.001), but higher Hb at day 42 (mean difference 0.49 g/dL [0.28, 0.69], *p* < 0.001; Table 2). In a sensitivity analysis, the removal of one study site at a time did not identify significant evidence of bias related to the included studies (Additional file 1: Table S10).

**Table 2:** The mean difference in haemoglobin between patients treated with and without primaquine

	Day of nadir		Day 7		Day 42	
Patient group	Mean difference* (95% CI, g/dL)	*p* value	Mean difference* (95% CI, g/dL)	*p* value	Mean difference* (95% CI, g/dL)	*p* value
Overall (*n* = 3421^†^)	− 0.13 (− 0.27, 0.01)	0.072	− 0.34 (− 0.46, − 0.23)	< 0.001	0.49 (0.28, 0.69)	< 0.001
Normal G6PD status (*n* = 1692)	0.05 (− 0.14, 0.24)	0.577	− 0.23 (− 0.39, − 0.07)	0.004	0.89 (0.53, 1.26)	< 0.001
Unknown G6PD status (*n* = 1701)	− 0.65 (− 0.82, − 0.47)	< 0.001	− 0.44 (− 0.66, − 0.21)	< 0.001	Not calculable^‡^	–
Females (*n* = 1210)	− 0.25 (− 0.43, − 0.07)	0.007	− 0.36 (− 0.54, − 0.18)	< 0.001	0.45 (0.18, 0.72)	0.001
Males (*n* = 2211)	− 0.11 (− 0.30, 0.08)	0.241	− 0.33 (− 0.48, − 0.18)	< 0.001	0.62 (0.29, 0.94)	< 0.001

*CI* confidence interval

*The difference in the mean haemoglobin comparing patients treated with or without primaquine. A negative mean difference equates to a lower haemoglobin when treated with chloroquine plus primaquine. Linear mixed effects models with non-linear terms for time were used to derive estimates of mean haemoglobin at day of nadir, day 7 and day 42

†*n* represents the number of patients who contributed at least one follow-up haemoglobin measurement for the linear mixed effects modelling of the haemoglobin trajectories

‡no day 42 haemoglobin measurements were available for patients treated with chloroquine plus primaquine

Of the 1446 patients treated with PQ, 38.2% (553) were treated with a high daily dose and 61.8% (893) with a low daily dose. There was no significant difference in mean Hb between patients treated with a high or low daily PQ dose, either at day 3 (mean difference 0.14 g/dL [− 0.05, 0.33], *p* = 0.161) or day 7 (mean difference 0.18 g/dL [− 0.11, 0.46], *p* = 0.227).

In subgroup analyses, the mean Hb at the day of nadir was significantly lower in patients treated with CQ+PQ than in those treated with CQ in females (mean difference − 0.25 g/dL [− 0.43, − 0.07], *p* = 0.007), and patients with unknown G6PD status (mean difference – 0.65 g/dL [− 0.82, − 0.47], *p* < 0.001), but there was no significant difference between treatment groups in males or patients known to be G6PD normal (Table 2). In G6PD normal patients, the following factors were associated with a greater reduction in Hb at day of nadir: younger age, higher baseline Hb, higher baseline parasitaemia, female gender and short relapse periodicity (Additional file 1: Table S11). By day 42, the mean Hb was higher following CQ+PQ compared to CQ alone for females (mean difference 0.45 g/dL [0.18, 0.72], *p* = 0.001), males (0.62 g/dL [0.29, 0.94], *p* < 0.001) and patients with normal G6PD status (0.89 g/dL [0.53, 1.26], *p* < 0.001) (Fig. 2 and Additional file 1: Figure S3). None of the patients with unknown G6PD status treated with CQ+PQ had a Hb measure at day 42, precluding day 42 comparison between treatment groups in this subgroup.

Overall, 17.4% (344/1975) of patients treated with CQ had recurrent parasitaemia between days 7 and 42, compared to 2.0% (29/1446) of those treated with CQ+PQ. The mean Hb at day 42 was significantly lower in patients with recurrent parasitaemia compared to those with no recurrence (mean difference − 0.72 g/dL [− 0.90, − 0.54], *p* < 0.001). The only G6PD-deficient patient treated with CQ+PQ had a haemoglobin fall from 14 g/dL at day 0 to 6.6 g/dL at day 14 but was not tested in between (Additional file 1: Table S12-S13).

### Effect of delayed parasite clearance on haemoglobin profile

In total, 37.1% (1000/2698) of patients had cleared their parasitaemia by day 1, 76.9% (2076/2698) had cleared by day 2 and 23.1% (622/2698) had parasite clearance delayed until after day 2. The proportion with delayed parasite clearance after day 2 was 17.6% (290/1646) following CQ and 31.6% (332/1052) following CQ+PQ. After controlling for confounding factors including PQ treatment, patients with delayed parasite clearance had a significantly lower Hb at the day of nadir (mean difference − 0.26 g/dL [− 0.45, − 0.06], *p* = 0.010) and day 42 (mean difference − 0.23 g/dL [− 0.39, − 0.07], *p* = 0.004) (Additional file 1: Figure S4).

### Safety outcomes

None of the patients died. Whilst 1.1% (7/610) of patients treated with CQ and 5.7% (27/471) treated with CQ+PQ had a fractional fall in Hb greater than 25% from baseline at day 2/3, 94.1% (32/34) of these patients started with a Hb greater than or equal to 11.5 g/dl. On day 2/3, none of the 610 patients treated with CQ alone had a clinically significant fall (fall in Hb > 25% to < 7 g/dL) or a fall greater than 5 g/dL. Of the patients treated with CQ+PQ, one G6PD normal male patient had a clinically significant fall and six patients with G6PD unknown status had a fall greater than 5 g/dL, one of whom was female (Table 3 and Additional file 1: Table S12–S13). On day 7, G6PD normal patients had a 0.3% (1/389) risk of clinically significant haemolysis and a 1% (4/389) risk of a fall in haemoglobin > 5 g/dL. The risks of safety outcomes occurring at day 28 and in patients with unknown or deficient G6PD status are presented in Table 3, Fig. 4 and Additional file 1: Table S14. No patients were reported to have received a blood transfusion.

**Table 3:** Distribution of absolute and percentage change in haemoglobin and risk of anaemia by the treatment group and G6PD status

	Any G6PD status		Normal G6PD status		Unknown G6PD status	
Day and metric	Chloroquine alone	Chloroquine plus primaquine	Chloroquine alone	Chloroquine plus primaquine	Chloroquine alone	Chloroquine plus primaquine
Day 2/3 (number of patients)	610*	471^†^	338	334	258	137
Absolute change^‡^, mean (SD) [range]; g/dL	− 0.5 (1.1) [− 4.6 to 3.4]	− 1.1 (1.6) [− 6.4 to 5.0]	− 0.9 (1.2) [− 4.6 to 3.4]	− 1.2 (1.3) [− 4.2 to 5.0]	0.1 (0.6) [− 2.1 to 2.8]	− 1.0 (2.1) [− 6.4 to 3.8]
Percentage change^‡^, mean (SD) [range]; %	− 3.5 (8.5) [− 32.3 to 34.3]	− 8.0 (11.8) [− 39.4 to 60.8]	− 6.7 (9.0) [− 32.3 to 34.3]	− 8.8 (10.1) [− 39.4 to 60.8]	0.9 (5.4) [− 17.1 to 27.5]	− 6.2 (14.9) [− 35.8 to 35.5]
Percentage fall > 25%	7/610 (1.1%)	27/471 (5.7%)	7/338 (2.1%)	11/334 (3.3%)	0/258 (0%)	16/137 (11.7%)
> 25% fall associated with severe anaemia (%)^§^	0/610 (0%)	1/471 (0.2%)	0/338 (0%)	1/334 (0.3%)	0/258 (0%)	0/137 (0%)
Absolute fall > 5 g/dL^¶^	0/610 (0%)	6/471 (1.3%)	0/338 (0%)	0/334 (0%)	0/258 (0%)	6/137 (4.4%)
Day 7 ± 2	1222	539	608	389	588	150
Absolute change^‡^, mean (SD) [range]; g/dL	− 0.1 (1.2) [− 5.6 to 6.5]	− 1.0 (1.6) [− 7.3 to 5.4]	− 0.2 (1.4) [− 4.6 to 6.5]	− 1.0 (1.5) [− 7.3 to 5.4]	0.1 (1.1) [− 5.6 to 5.4]	− 0.8 (2.0) [− 6.0 to 3.8]
Percentage change^‡^, mean (SD) [range]; %	0.4 (10.5) [− 40.6 to 64.4]	− 6.5 (12.3) [− 55.3 to 65.5]	− 0.7 (11.3) [− 33.0 to 64.4]	− 7.3 (11.5) [− 55.3 to 65.5]	1.7 (9.4) [− 40.6 to 52.4]	− 6.2 (14.9) [− 35.8 to 35.5]
Percentage fall > 25%	5/1222 (0.4%)	33/539 (6.1%)	4/608 (0.7%)	20/389 (5.1%)	1/588 (0.2%)	13/150 (8.7%)
> 25% fall associated with severe anaemia (%)^§^	0/1220 (0%)	1/539 (0.2%)	0/608 (0%)	1/389 (0.3%)	0/586 (0%)	0/150 (0%)
Absolute fall > 5 g/dL^¶^	1/1222 (0.1%)	8/539 (1.5%)	0/608 (0%)	4/389 (1.0%)	1/588 (0.2%)	4/150 (2.7%)
Day 28 ± 3	1579	917	731	472	826	444
Absolute change^‡^, mean (SD) [range]; g/dL	0.5 (1.4) [− 6.9 to 6.2]	0.4 (1.7) [− 6.8 to 6.7]	0.4 (1.5) [− 4.7 to 6.2]	0.4 (1.4) [− 4.2 to 6.2]	0.5 (1.4) [− 6.9 to 6.0]	0.5 (1.9) [− 6.8 to 6.7]
Percentage change^‡^, mean (SD) [range]; %	5.0 (13.4) [− 46.3 to 81.7]	5.1 (16.5) [− 51.1 to 136.7]	4.2 (13.1) [− 39.5 to 74.7]	4.2 (12.8) [− 36.2 to 74.8]	5.8 (13.7) [− 46.3 to 81.7]	6.1 (19.7) [− 51.1 to 136.7]
Percentage fall > 25%	9/1579 (0.6%)	13/917 (1.4%)	6/731 (0.8%)	2/472 (0.4%)	3/826 (0.4%)	11/444 (2.5%)
> 25% fall associated with severe anaemia (%)^§^	1/1576 (0.1%)	3/906 (0.3%)	0/730 (0%)	0/472 (0%)	1/824 (0.1%)	3/433 (0.7%)
Absolute fall > 5 g/dL^¶^	1/1579 (0.1%)	3/917 (0.3%)	0/731 (0%)	0/472 (0%)	1/826 (0.1%)	3/444 (0.7%)

*CI* confidence interval, *Hb* haemoglobin, *No* number, *SD* standard deviation

*Includes 338 patients with normal G6PD status, 258 with unknown status and 14 with borderline or deficient status

^†^Includes 334 patients with normal G6PD status and 137 with unknown status

^‡^Results are reported as a change in haemoglobin, with positive results reflecting a rise in Hb and negative results reflecting a fall in Hb

^§^Patients were considered to develop severe anaemia if their baseline Hb was ≥ 7 g/dL and their follow-up Hb was < 7 g/dL, with the denominator the number of people with a Hb recorded for that day who had a baseline ≥ 7 g/dL. All patients that developed severe anaemia had a Hb fall > 25%. Additional file 1: Table S12 provides additional patient details

^¶^Additional file 1: Table S13 provides additional patient details

In unadjusted analyses of G6PD normal patients, the number needed to harm to have a clinically significant drop in Hb at day 2/3 was 334 exposures to PQ and the corresponding number needed to harm at day 7 was 389 patients.

## Discussion

This meta-analysis of data from 3421 individual patients enrolled in 29 studies provides the most detailed evaluation of the haematological consequences of vivax malaria treated with CQ, with and without PQ, in over 60 years. In patients with normal G6PD status, patients treated with PQ had no additional clinically significant haemolysis compared to CQ alone. However, patients treated with PQ had higher haemoglobins by day 42 (0.5 g/dL higher), a difference likely attributable in part to a reduction in recurrent parasitaemia.

Treatment with PQ reduces the risk of vivax recurrences at day 42 by up to 90%, predominantly because of its ability to prevent reactivation of dormant liver stages [47, 49, 52, 53]. Despite this benefit, clinician concern regarding the risk of severe haemolysis in patients with G6PDd, coupled with a lack of reliable point of care tests for G6PDd, has prevented the widespread uptake of PQ radical cure in many vivax-endemic regions [12]. The risks of severe haemolysis attributable to PQ need to be quantified and weighed against the underlying risk of anaemia attributable to malaria itself. Our analysis highlights that in a study population where the majority of patients were confirmed or suspected to be G6PD normal, there was minimal additional haemolysis attributable to PQ beyond the fall in Hb occurring after treatment with CQ. In our analysis, the fall in Hb was not influenced by the daily dose of PQ administered. Consistent with previous studies, by day 42, patients treated with PQ had a substantially higher Hb, likely reflecting the prevention of relapse and potential recrudescence [49, 54].

Previous antimalarial studies have used an arbitrary fall in Hb of > 25% as a safety outcome [55, 56]. Whilst 5.7% treated with CQ+PQ had a fractional fall in Hb > 25% at day 2/3, almost all of these patients had a high baseline Hb; hence, a large fractional fall in Hb may not necessarily equate to clinically relevant morbidity. We explored two alternative clinically specific safety measures: a composite measure of a fall > 25% from baseline to a Hb < 7 g/dL, and a fall in Hb > 5 g/dL. The former reflects Hb reduction to a level associated with rising risk of mortality [6], and the latter possible substantial intravascular haemolysis leading to an increased risk of high cell-free Hb and acute renal failure [57]. The overall risk of these safety outcomes at day 2/3 was approximately 15 per 1000 patients treated, substantially less than the risk of patients having a high fractional fall in Hb.

Reassuringly in this study population, no patients with unknown G6PD status had a clinically relevant fall in Hb at either day 2/3 or day 7. These patients were from Brazil, Indonesia and Vietnam, where G6PD testing is not routinely recommended in the National guidelines. Although these study populations may be perceived to have a low risk of G6PDd, these areas are known to include some patients with severe G6PD variants. In the studies included in our analysis when patients were screened for G6PDd, normal activity was defined as an enzyme activity greater than 30%. This approach will not have excluded heterozygous females with intermediate G6PD activity and is the likely reason that the mean nadir Hb of female patients was lower following CQ+PQ compared to females treated with CQ alone. Importantly, only one female patient had a clinically relevant fall in Hb at day 2/3 or day 7. In the G6PD normal patients, PQ treatment was associated with one additional patient with a clinically relevant fall in Hb at day 2/3 for every 334 patients treated; these estimates are unadjusted for confounding factors. Hence, whilst PQ did not cause a significant increase in the population risk of haemolysis in patients with proven or suspected G6PD normal status, there remains an appreciable risk of severe haemolysis in vulnerable individuals. Our study highlights the importance of reliable and accurate point of care testing of G6PD status prior to radical cure of *P. vivax*, in conjunction with clinical or laboratory monitoring for haematological recovery.

The day of nadir Hb occurred on day 2 in patients treated with CQ alone and day 3 in those treated with CQ+PQ, and yet less than half of the clinical studies sampled Hb on these days routinely. Future studies aiming to quantify PQ-induced haemolysis should consider reviewing patients around day 3, after completion of blood schizontocidal treatment, at which time patients at greatest risk of clinically harmful haemolysis could be identified and appropriate management initiated, if indicated.

Our analysis included patients irrespective of their G6PD status, all of whom were judged to be suitable for treatment with PQ. Not all studies tested patients for G6PDd, reflecting variations in regional protocols. Patients with unknown G6PD status treated with PQ had a lower mean Hb at nadir which may reflect unidentified patients with G6PDd. Furthermore, even in female patients with normal G6PD status, those treated with PQ had a lower mean Hb at nadir which may reflect inclusion of heterozygous individuals with intermediate G6PDd who would have been screened as G6PD normal by qualitative tests. A small number of adverse safety outcomes occurred with and without primaquine treatment across all patient groups; within the first 7 days, 53% (8/15) of adverse events occurred in patients with normal G6PD status, 47% (7/15) in those with unknown status and 27% (4/15) in female patients.

Our study has several important limitations. Lack of PQ randomisation led to the potential for differences between patient groups and selection bias that could not be adjusted for. For example, no patients from Africa were treated with PQ. Inclusion of data from only half of the patients from the targeted clinical trials is an additional limitation. Despite minor epidemiological differences between the populations of studies included and targeted (Additional file 1: Table S8), the studies in our analysis were undertaken in a range of populations in vivax-endemic areas. Furthermore, the mean baseline Hb was similar between the included and targeted studies suggesting that differences in the haematological profiles of these populations were unlikely to be an important source of bias. A sensitivity analysis did not identify significant evidence of bias related to the included studies (Additional file 1: Table S10). Whilst it is likely that our findings can be generalised to G6PD normal patients in many vivax-endemic regions, the variation in G6PD variants across different regions and the disproportionate number of male patients in the current study prevent the overall estimates of risk including patients with unknown G6PD status from being generalised globally. Finally, restriction of follow-up to a maximum of 42 days prevented us from being able to estimate the overall haematological benefit beyond day 42 related to prevention of multiple future relapses as opposed to just the first relapse.

## Conclusions

In summary, PQ administration in G6PD normal patients was not associated with a greater acute fall in Hb compared to patients not treated with PQ. The reduction in Hb after treatment for vivax malaria was primarily associated with the disease itself rather than haemolysis due to PQ treatment. Indeed, within 42 days, patients treated with PQ had better haematological outcomes than those treated with chloroquine alone, consistent with the prevention of further haematological insults caused by recurrent parasitaemia. There was a small but clinically relevant risk of severe Hb reduction after treatment with PQ, even in patients with normal G6PD status. Our results highlight the public health benefits of radical cure for the treatment of *P. vivax* when this can be offered in combination with accurate point of care testing for G6PDd.

## Additional file

Additional file 1: **Checklist 1.** PRISMA-IPD. **Box 1.** Search strategy. **Table S1.** Studies included in the analysis. **Table S2.** Reasons for studies not being included in the analysis. **Table S3.** Studies targeted for the analysis but not included. **Table S4.** Country of origin and background prevalence of G6PD deficiency in patients with unknown G6PD status. **Table S5.** Planned primaquine regimens. **Figure S1.** Study sites for clinical trials. **Table S6.** Demographics, baseline characteristics and baseline haemoglobin measurements of G6PD normal patients. **Table S7.** Demographics, baseline characteristics and baseline haemoglobin measurements of patients with unknown G6PD status. **Table S8.** Comparison of baseline characteristics between included and targeted studies. **Table S9.** Risk factors for baseline anaemia (Hb < 10 g/dL). **Figure S2.** Relationship between day 0 haemoglobin and percentage and absolute change in haemoglobin on day 2/3. **Table S10.** Sensitivity analysis for change in haemoglobin for patients treated with chloroquine compared to chloroquine and primaquine. **Table S11.** Factors associated with change in haemoglobin between day 0 and day 2/3 in G6PD normal patients. **Figure S3.** Mean haemoglobin versus time profiles for female and male patients treated with chloroquine with or without primaquine. **Table S12.** Patients with a Hb fall > 25% leading to development of severe anaemia (Hb < 7 g/dL) during the first 42 days. **Table S13.** Patients with haemoglobin falling > 5 g/dL during the first 42 days. **Figure S4.** Mean haemoglobin versus time profile for patients with or without delayed parasite clearance. **Table S14.** Unadjusted absolute and percentage change in haemoglobin and risk of anaemia if G6PD deficient. **References S1.** Studies not included in the analysis. (PDF 1347 kb)

## Data Availability

The data that support the findings of this study are available for access via the WorldWide Antimalarial Resistance Network (WWARN.org). Requests for access will be reviewed by a Data Access Committee to ensure that the use of data protects the interests of the participants and researchers according to the terms of ethics approval and principles of equitable data sharing. Requests can be submitted by email to malariaDAC@iddo.org via the Data Access Form available at WWARN.org/accessing-data. The WWARN is registered with the Registry of Research Data Repositories (re3data.org).

## Abbreviations

AOR: Adjusted odds ratio
CI: Confidence interval
CQ: Chloroquine
G6PD: Glucose-6-phosphate dehydrogenase
G6PDd: Glucose-6-phosphate dehydrogenase deficiency
Hb: Haemoglobin
IQR: Inter-quartile range
PQ: Primaquine
PRISMA: Preferred Reporting Items for Systematic Reviews and Meta-Analyses
PROSPERO: International Prospective Register of Systematic Reviews
RBC: Red blood cells
SD: Standard deviation
WWARN: WorldWide Antimalarial Resistance Network
