# Supplementary material for: The haematological consequences of Plasmodium vivax malaria after chloroquine treatment with and without primaquine: a WorldWide Antimalarial Resistance Network systematic review and individual patient data meta-analysis
Source: BMC Med. 2019 Aug 1;17:151. doi: 10.1186/s12916-019-1386-6 (PMC6670141; doi:10.1186/s12916-019-1386-6)
Supplement: Supplementary file 1 — Checklist 1. PRISMA-IPD. Box 1. Search strategy. Table S1. Studies included in the analysis. Table S2. Reasons for studies not being included in the analysis. Table S3. Studies targeted for the analysis but not included. Table S4. Country of origin and background prevalence of G6PD deficiency in patients with unknown G6PD status. Table S5. Planned primaquine regimens. Figure S1. Study sites for clinical trials. Table S6. Demographics, baseline characteristics and baseline haemoglobin measurements of G6PD normal patients. Table S7. Demographics, baseline characteristics and baseline haemoglobin measurements of patients with unknown G6PD status. Table S8. Comparison of baseline characteristics between included and targeted studies. Table S9. Risk factors for baseline anaemia (Hb < 10 g/dL). Figure S2. Relationship between day 0 haemoglobin and percentage and absolute change in haemoglobin on day 2/3. Table S10. Sensitivity analysis for change in haemoglobin for patients treated with chloroquine compared to chloroquine and primaquine. Table S11. Factors associated with change in haemoglobin between day 0 and day 2/3 in G6PD normal patients. Figure S3. Mean haemoglobin versus time profiles for female and male patients treated with chloroquine with or without primaquine. Table S12. Patients with a Hb fall > 25% leading to development of severe anaemia (Hb < 7 g/dL) during the first 42 days. Table S13. Patients with haemoglobin falling > 5 g/dL during the first 42 days. Figure S4. Mean haemoglobin versus time profile for patients with or without delayed parasite clearance. Table S14. Unadjusted absolute and percentage change in haemoglobin and risk of anaemia if G6PD deficient. References S1. Studies not included in the analysis. (PDF 1347 kb) [file 12916_2019_1386_MOESM1_ESM.pdf]

ADDITIONAL FILE 1

Commons *et al*, The haematological consequences of *Plasmodium vivax* malaria after chloroquine treatment with or without primaquine: a WorldWide Antimalarial Resistance Network systematic review and individual patient data meta-analysis

	Page
**Checklist S1.** PRISMA-IPD	2
**Box S1.** Search Strategy	6
**Table S1.** Studies included in analysis	7
**Table S2.** Reasons for studies not being included in analysis	9
**Table S3.** Studies targeted for the analysis but not included	10
**Table S4.** Country of origin and background prevalence of G6PD deficiency in patients with unknown G6PD status	12
**Table S5.** Planned primaquine regimens	13
**Figure S1.** Study sites for clinical trial	14
**Table S6.** Demographics, baseline characteristics and baseline haemoglobin measurements of G6PD normal patients	15
**Table S7.** Demographics, baseline characteristics and baseline haemoglobin measurements of patients with unknown G6PD status	16
**Table S8.** Comparison of baseline characteristics between included and targeted studies	17
**Table S9.** Risk factors for baseline anaemia (Hb < 10 g/dL)	18
**Figure S2.** Relationship between day 0 haemoglobin and percentage and absolute change in haemoglobin on day 2/3	19
**Table S10.** Sensitivity analysis for change in haemoglobin for patients treated with chloroquine compared to chloroquine and primaquine	20
**Table S11.** Factors associated with change in haemoglobin between day 0 and day 2/3 in G6PD normal patients	21
**Figure S3.** Mean haemoglobin versus time profiles for female and male patients treated with chloroquine with or without primaquine	22
**Table S12.** Patients with a Hb fall >25% leading to development of severe anaemia (Hb <7 g/dL) during the first 42 days	23
**Table S13.** Patients with haemoglobin falling >5 g/dL during the first 42 days	24
**Figure S4.** Mean haemoglobin versus time profile for patients with or without delayed parasite clearance	25
**Table S14.** Unadjusted absolute and percentage change in haemoglobin and risk of anaemia if G6PD status unknown or deficient	26
**References S1.** Studies not included in analysis	27

Checklist S1. PRISMA-IPD Checklist of items to include when reporting a systematic review and meta-analysis of individual participant data (IPD)

PRISMA-IPD Section/topic	Item No	Checklist item	Reported on page
**Title**			
Title	1	Identify the report as a systematic review and meta-analysis of individual participant data.	1
**Abstract**			
Structured summary	2	Provide a structured summary including as applicable:	6
		**Background**: state research question and main objectives, with information on participants, interventions, comparators and outcomes.	6
		**Methods**: report eligibility criteria; data sources including dates of last bibliographic search or elicitation, noting that IPD were sought; methods of assessing risk of bias.	6
		**Results**: provide number and type of studies and participants identified and number (%) obtained; summary effect estimates for main outcomes (benefits and harms) with confidence intervals and measures of statistical heterogeneity. Describe the direction and size of summary effects in terms meaningful to those who would put findings into practice.	6
		**Discussion:** state main strengths and limitations of the evidence, general interpretation of the results and any important implications.	7
		**Other:** report primary funding source, registration number and registry name for the systematic review and IPD meta-analysis.	7
**Introduction**			
Rationale	3	Describe the rationale for the review in the context of what is already known.	8
Objectives	4	Provide an explicit statement of the questions being addressed with reference, as applicable, to participants, interventions, comparisons, outcomes and study design (PICOS). Include any hypotheses that relate to particular types of participant-level subgroups.	8
**Methods**			
Protocol and registration	5	Indicate if a protocol exists and where it can be accessed. If available, provide registration information including registration number and registry name. Provide publication details, if applicable.	9
Eligibility criteria	6	Specify inclusion and exclusion criteria including those relating to participants, interventions, comparisons, outcomes, study design and characteristics (e.g. years when conducted, required minimum follow-up). Note whether these were applied at the study or individual level i.e. whether eligible participants were included (and ineligible participants excluded) from a study that included a wider population than specified by the review inclusion criteria. The rationale for criteria should be stated.	9-10
Identifying studies - information sources	7	Describe all methods of identifying published and unpublished studies including, as applicable: which bibliographic databases were searched with dates of coverage; details of any hand searching including of conference proceedings; use of study registers and agency or company databases; contact with the original research team and experts in the field; open adverts and surveys. Give the date of last search or elicitation.	9
Identifying studies - search	8	Present the full electronic search strategy for at least one database, including any limits used, such that it could be repeated.	Additional file 1

Study selection processes	9	State the process for determining which studies were eligible for inclusion.	9-10
Data collection processes	10	Describe how IPD were requested, collected and managed, including any processes for querying and confirming data with investigators. If IPD were not sought from any eligible study, the reason for this should be stated (for each such study).	9; Ref 16
		If applicable, describe how any studies for which IPD were not available were dealt with. This should include whether, how and what aggregate data were sought or extracted from study reports and publications (such as extracting data independently in duplicate) and any processes for obtaining and confirming these data with investigators.	Additional file 1
Data items	11	Describe how the information and variables to be collected were chosen. List and define all study level and participant level data that were sought, including baseline and follow-up information. If applicable, describe methods of standardising or translating variables within the IPD datasets to ensure common scales or measurements across studies.	9-10
IPD integrity	A1	Describe what aspects of IPD were subject to data checking (such as sequence generation, data consistency and completeness, baseline imbalance) and how this was done.	9; Ref 16
Risk of bias assessment in individual studies.	12	Describe methods used to assess risk of bias in the individual studies and whether this was applied separately for each outcome. If applicable, describe how findings of IPD checking were used to inform the assessment. Report if and how risk of bias assessment was used in any data synthesis.	11
Specification of outcomes and effect measures	13	State all treatment comparisons of interests. State all outcomes addressed and define them in detail. State whether they were pre-specified for the review and, if applicable, whether they were primary/main or secondary/additional outcomes. Give the principal measures of effect (such as risk ratio, hazard ratio, difference in means) used for each outcome.	11-12
Synthesis methods	14	Describe the meta-analysis methods used to synthesise IPD. Specify any statistical methods and models used. Issues should include (but are not restricted to):<br>• Use of a one-stage or two-stage approach.<br>• How effect estimates were generated separately within each study and combined across studies (where applicable).<br>• Specification of one-stage models (where applicable) including how clustering of patients within studies was accounted for.<br>• Use of fixed or random effects models and any other model assumptions, such as proportional hazards.<br>• How (summary) survival curves were generated (where applicable).<br>• Methods for quantifying statistical heterogeneity (such as I^2 and τ^2).<br>• How studies providing IPD and not providing IPD were analysed together (where applicable).<br>• How missing data within the IPD were dealt with (where applicable).	11-12; Ref 16
Exploration of variation in effects	A2	If applicable, describe any methods used to explore variation in effects by study or participant level characteristics (such as estimation of interactions between effect and covariates). State all participant-level characteristics that were analysed as potential effect modifiers, and whether these were pre-specified.	11
Risk of bias across studies	15	Specify any assessment of risk of bias relating to the accumulated body of evidence, including any pertaining to not obtaining IPD for particular studies, outcomes or other variables.	11

Additional analyses	16	Describe methods of any additional analyses, including sensitivity analyses. State which of these were pre-specified.	11-12
**Results**			
Study selection and IPD obtained	17	Give numbers of studies screened, assessed for eligibility, and included in the systematic review with reasons for exclusions at each stage. Indicate the number of studies and participants for which IPD were sought and for which IPD were obtained. For those studies where IPD were not available, give the numbers of studies and participants for which aggregate data were available. Report reasons for non-availability of IPD. Include a flow diagram.	12; Fig 1
Study characteristics	18	For each study, present information on key study and participant characteristics (such as description of interventions, numbers of participants, demographic data, unavailability of outcomes, funding source, and if applicable duration of follow-up). Provide (main) citations for each study. Where applicable, also report similar study characteristics for any studies not providing IPD.	Additional file 1
IPD integrity	A3	Report any important issues identified in checking IPD or state that there were none.	12; Fig 1
Risk of bias within studies	19	Present data on risk of bias assessments. If applicable, describe whether data checking led to the up-weighting or down-weighting of these assessments. Consider how any potential bias impacts on the robustness of meta-analysis conclusions.	13; Additional file 1
Results of individual studies	20	For each comparison and for each main outcome (benefit or harm), for each individual study report the number of eligible participants for which data were obtained and show simple summary data for each intervention group (including, where applicable, the number of events), effect estimates and confidence intervals. These may be tabulated or included on a forest plot.	Additional file 1
Results of syntheses	21	Present summary effects for each meta-analysis undertaken, including confidence intervals and measures of statistical heterogeneity. State whether the analysis was pre-specified, and report the numbers of studies and participants and, where applicable, the number of events on which it is based.	15-18
		When exploring variation in effects due to patient or study characteristics, present summary interaction estimates for each characteristic examined, including confidence intervals and measures of statistical heterogeneity. State whether the analysis was pre-specified. State whether any interaction is consistent across trials.	
		Provide a description of the direction and size of effect in terms meaningful to those who would put findings into practice.	
Risk of bias across studies	22	Present results of any assessment of risk of bias relating to the accumulated body of evidence, including any pertaining to the availability and representativeness of available studies, outcomes or other variables.	12-13; Additional file 1
Additional analyses	23	Give results of any additional analyses (e.g. sensitivity analyses). If applicable, this should also include any analyses that incorporate aggregate data for studies that do not have IPD. If applicable, summarise the main meta-analysis results following the inclusion or exclusion of studies for which IPD were not available.	15-20; Additional file 1
**Discussion**			
Summary of evidence	24	Summarise the main findings, including the strength of evidence for each main outcome.	23-26

Strengths and limitations	25	Discuss any important strengths and limitations of the evidence including the benefits of access to IPD and any limitations arising from IPD that were not available.	25-26
Conclusions	26	Provide a general interpretation of the findings in the context of other evidence.	26
Implications	A4	Consider relevance to key groups (such as policy makers, service providers and service users). Consider implications for future research.	223-26
**Funding**			
Funding	27	Describe sources of funding and other support (such as supply of IPD), and the role in the systematic review of those providing such support.	29

© Reproduced with permission of the PRISMA IPD Group, which encourages sharing and reuse for non-commercial purposes

Box S1. Search strategy

Search strategy

All prospective *P. vivax* antimalarial clinical trials published between Jan 1, 2000 and March 22, 2017 were identified by the application of the key terms (listed below) through Medline (Pubmed), Web of Science, Embase and the Cochrane Database of Systematic Reviews. Abstracts of all references containing any mention of antimalarial drugs were manually checked to confirm prospective clinical trials, with review of full text when needed. Studies on prevention, prophylaxis, reviews, animal studies, patients with severe malaria, where schizontocidal treatment was unsupervised or where data were extracted retrospectively from medical records outside of a planned trial were excluded. The review process is documented in more detail in Commons *et al*, Int J Parasitol Drug Drug Res 2017 [15]. The year of the study was taken as the year in which the paper was published, although the start and end date of patient enrolment were also recorded.

Key terms

Literature search (conducted March 2017) with the following key terms (version undertaken in Pubmed): (malaria OR plasmod*) AND (amodiaquine OR atovaquone OR artemisinin OR arteether OR artesunate OR artemether OR artemotil OR azithromycin OR artekin OR chloroquine OR chlorproguanil OR cycloguanil OR clindamycin OR coartem OR dapsone OR dihydroartemisinin OR duo-cotecxin OR doxycycline OR halofantrine OR lumefantrine OR lariam OR malarone OR mefloquine OR naphthoquine OR naphthoquinone OR piperaquine OR primaquine OR proguanil OR pyrimethamine OR pyronaridine OR proguanil OR quinidine OR quinine OR riamet OR sulphadoxine OR tetracycline OR tafenoquine).

Table S1. Studies included in analysis

Author-year	Country	Recruitment period	Relapse periodicity*	Study sites	Age range, years	Follow up, days	Treatment arms	Drug supervision	Drug origin	Patients enrolled in initial study	Patient data available	Patients included in current study	G6PD tested at screening	Baseline Hb, g/dL (mean (SD))	Management of patients with anaemia	PQ dosing	Timing of PQ	Published
Taylor-2001 [23]	Indonesia	1995-1998	Short	1	16-41	28	CQ, CQ+Doxy, Doxy	Full	CQ: Dumex, Indonesia	63	64	23	No	13·0 (2·1)	No haematinics given	14day-High Dose	End of Study	Yes
Phan-2002 [24]	Vietnam	1997-2000	Short	1	19-51	28	CQ, Art	Full	CQ: Mekophar Company, Vietnam	226	232	7	No	12·6 (1·1)	Not standardised	5day-Very Low Dose	End of Study	Yes
Leslie-2007 [25]	Afghanistan, Pakistan	2004-2006	Long	2	4-63	28	CQ, Chlorproguanil-dapsone, SP	Full		767	767	159	Yes Quantitative (Spectrophotometry)	12·6 (1·9)	Not standardised	-	-	Yes
Ratcliff-2007 [26]	Indonesia	2004-2005	Short	1	1-60	28	CQ	Full	CQ: P.T. Bayer, Indonesia	40	61	33	No	10·4 (2·3)	Not standardised	14day-Low Dose	End of Study	Yes
Leslie-2008 [27]	Pakistan	2004-2007	Long	3	4-50	365	CQ, CQ+PQ, CQ+PQ	Full		200	210	122	Yes Quantitative (Spectrophotometry)	12·3 (1·8)	Not standardised	None, 14day-High Dose, weekly over 8 weeks (excluded)	Day0	Yes
Ketema-2009 [28]	Ethiopia	2007-2008	Long	1	0-45	28	CQ	Full	CQ: Adigrat Pharmaceutical Factory, Ethiopia	84	84	83	No	12·1 (2·0)	No haematinics given	-	-	Yes
Awab-2010 [29]	Afghanistan	2007-2009	Long	3	0-71	56	CQ, DP	Full	CQ: IDA, Netherlands	536	536	265	No	10·9 (1·5)	Not standardised	-	-	Yes
Phyo-2011 [30]	Thailand	2007-2008	Short	1	1-63	63	CQ, DP	Full	CQ: Government Pharmaceutical Organization, Thailand	492	498	243	Yes Qualitative (FST)	12·3 (2·0)	Treated if Hct <30%: >13 years ferrous sulphate + folate Children >10kg ferrous sulphate + folate; Children <10kg ferrous fumerate	14day-High Dose	End of Study	Yes
Poravuth-2011 [31]	Multicentred	2007-2008	Variable	5	17-51	42	CQ, AS+Pyr	Full	CQ: Shin Poong Pharmaceutical Co, Ltd, Korea	456	456	30	Yes Qualitative (FST)	12·2 (2·1)	Not standardised	14day-Low Dose	Day28	Yes
Barber-2013 [32]	Malaysia	2010-2011	Short	1	20-49	43	CQ+PQ, varied	Not stated		43	86	3	Yes Qualitative (FST)	12·9 (0·8)	Not standardised	14day-High Dose	Day0	Yes
Hwang-2013 [33]	Ethiopia	2009-2010	Long	2	1-65	42	CQ, AL	Full CQ; Partial AL	CQ: AralenH, Sanofi-Aventis, US	242	242	120	No	13·0 (2·5)	Treated if Hb <10g/dL: Ferrous sulphate, folate + mebendazole (if >1 year)	-	-	Yes
Marques-2014 [34]	Brazil	2007-2008	Long	1	13-65	28	CQ+PQ	Full	Farmanguinhos, Brazil	135	154	135	No	13·9 (1·6)	No haematinics given	7 day-Low Dose	Day0	Yes
Anez-2015 [35]	Bolivia	2011	Long	1	5-61	28	CQ	Full	CQ: Macleods Pharmaceuticals Ltd, India	100	100	96	No	11·1 (1·8)	Treated with iron tablets	7 day-Low Dose	Day28	Yes
Getachew-2015 [36]	Ethiopia	2010-2013	Long	4	0-65	28	CQ	Full	CQ: Ethiopian Federal Ministry of Health	288	288	271	No	12·5 (2·2)	No haematinics given	-	-	Yes
Gomes-2015 [37]	Brazil	2011-2012	Long	1	10-56	28	CQ+PQ	Partial CQ and PQ	Coordination of Pharmaceutical Assistance of Amapá	103	94	92	No	12·7 (2·1)	No haematinics given	7day-Low Dose	Day0	Yes
Lidia-2015 [38]	Indonesia	2013	Short	1	18-88	42	CQ+PQ, DP+PQ	Full	CQ : Novapharin Pharmaceutical, Indonesia	51	51	26	No	10·2 (0·8)	No haematinics given	14day-Low Dose	Day0	Yes

									PQ: Phapros, Indonesia									
Rishikesh-2015 [39]	India	2012-2014	Long	1	18-76	28	CQ+PQ	Full CQ; Partial PQ	CQ: Bayer Pharmaceuticals, India PQ: IPCA Laboratories, India	125	125	124	Yes Quantitative (Spectrophotometry)	13·7 (2·2)	Not standardised	14day-Low Dose, weekly over 8 weeks (excluded)	Day0	Yes
Thanh-2015 [40]	Vietnam	2009-2011	Short	1	3-60	28	CQ+PQ	Full	National malaria control Program, Vietnam	260	260	260	No†	11·5 (2·2)	Treated if Hb ≤8g/dL: Ferrous sulphate + multivitamins	10day-High Dose	Day0	Yes
Grigg-2016 [41]	Malaysia	2012-2014	Short	3	9-35	42	AS+MQ+PQ, CQ+PQ	Full CQ and ASMQ Partial PQ	CQ: Kotra Pharma, Malaysia PQ: Pharmaniaga, Malaysia	103	103	8	Yes Qualitative (FST)	11·1 (1·5)	Not standardised	14day-High Dose	Day28	Yes
Ley-2016 [42]	Bangladesh	2014-2015	Short	1	6-30	30	AL+PQ, CQ+PQ	Partial AL; Full CQ; Partial PQ	CQ: Jayson Pharmaceuticals, Bangladesh PQ: Globe Pharmaceuticals, Bangladesh	55	66	12	Yes Quantitative (Spectrophotometry)	11·9 (2·6)	No haematinics given	14day-Low Dose	Day2	Yes
Pereira-2016 [43]	Brazil	2013-2015	Long	1	19-68	28	CQ+PQ	Partial CQ and PQ	Farmanguinhos—Fiocruz, Brazil	88	88	86	No	13·5 (1·5)	Not standardised	7-9day-Low Dose	Day0	Yes
Saravu-2016 [44]	India	2012-2015	Long	1	18-75	28	CQ+PQ, CQ+PQ(weekly)	Partial CQ and PQ	National Vector Borne Disease Control Programme, India	161	161	135	Yes Quantitative (Spectrophotometry)	13·2 (1·9)	Not standardised	14day-Low Dose, weekly over 8 weeks (excluded)	Day0	Yes
Thuan-2016 [45]	Vietnam	2013-2014	Short	2	7-67	63	CQ, DP	Full	CQ: National Malaria Program, Vietnam	128	128	44	Yes Qualitative (CareStart RDT)	13·5 (1·7)	No haematinics given.	14day-Low Dose	End of Study	Yes
Wangchuk-2016 [46]	Bhutan	2013-2015	Short	5	25-53	365	CQ+PQ	Full		24	28	4	No	12·8 (0·6)	Not standardised	14day-Low Dose	Day28	Yes
Abreha-2017 [47]	Ethiopia	2012-2016	Long	2	1-67	365	AL, AL+PQ, CQ, CQ+PQ	Partial AL; Full CQ; Partial PQ	CQ: Micro Labs Limited, India PQ: Sanofi-Aventis, US	398	398	102	Yes Qualitative (FST)	13·6 (1·7)	Not standardised	14day-Low Dose	Day2	After literature search
Chu-2018a [51]	Thailand	2012-2014	Short	1	1-61	365	DP+PQ, DP+PQ, CQ+PQ, CQ+PQ	Full	DP: Guilin Pharmaceutical, China PQ: Government Pharmaceutical Org, Thailand	680	680	337	Yes Qualitative (FST)	13·0 (2·0)	Treated if Hct <30%.	7day-High dose, 14day-High dose	Day0	After literature search
Grigg-2018 [50]	Malaysia	2013-2016	Short	1	11-29	42	CQ+PQ, variable	Full CQ; Partial PQ		57	57	3	Yes Qualitative (FST)	12·5 (0·4)	Not standardised	14day-High Dose	Day0	After literature search
Siqueira-2017 [48]	Brazil	2012-2013	Long	1	1-74	42	AS+AQ, CQ	Full	CQ: Farmanguinhos, Brazil	380	380	189	No	13·2 (1·7)	No haematinics given	-	-	Yes
Chu-2018b [49]	Thailand	2010	Short	1	1·5-63	365	CQ, CQ+PQ, AS	Full	CQ: Maneesh Pharmaceuticals & Medopharm, India PQ: Maneesh Pharmaceuticals, India & Government Pharmaceutical Organization, Thailand	645	645	409	Yes Qualitative (FST)	11·8 (1·7)	Treated if Hct <30%	None, 14day-High Dose	Day0	After literature search

Art – artemisinin; AS – artesunate; AL – artemether-lumefantrine; CQ – chloroquine; Doxy – doxycycline; DP – dihydroartemisinin-piperaquine; FST – fluorescent spot test; Hb – haemoglobin; Hct – haematocrit; PQ – primaquine; Pyr – pyronaridine; RDT – rapid diagnostic test; SD – standard deviation; SP – Sulfadoxine-pyrimethamine; * Short relapse periodicity ≤47 days; † One patient tested *post hoc*.

Table S2. Reasons for studies not being included in analysis

Reason	Number of studies	Studies*
No chloroquine treatment arm	28	58-85
Adjunctive drug(s) used	4	86-89
Study of pregnant women	2	90,91
No record of collecting haematological measurements in manuscript	70	92-161
Patient data available but no haematology results	8	162-169
Data not available	2	170,171
Investigators unable to be contacted	3	172-174
Missing minimum data for inclusion	3	175-177
Initial investigator response but no data provided	2	178,179
No response from investigators	21	55,180-199

* References of studies not included are provided in Additional File 1: References S1

Table S3. Studies targeted for the analysis but not included

Author-Year	Treatment arms	Sites	Country	Follow up, days	Randomised	Recruitment period	Treatment arms[*]	Patients treated with CQ+/-PQ	Haematology collected or reported in publication	Female (%)	Age		Haemoglobin		Haematocrit		G6PD status
											Mean (SD)	Median (range)	Mean (SD)	Median	Mean (SD)	Median	
Pukrittayakamee -2000 [180]	9	1	Thailand	28	Yes	1992-1998	CQ2+PQ14; CQ2; PQ14; Qu; Mfq; Halo; AS; Am; SP	60	Hct	0	25 (9)	Not stated	Not stated	Not stated	37 (-)[†]	Not stated	Not reported
Buchachart-2001 [181]	1	1	Thailand	28	No	1992-1997	CQ3+PQ14	593	Hct	37·1	25 (-)[†]	Not stated	Not stated	Not stated	36·2 (-)	Not stated	34 deficient 559 normal
Fryauff-2002 [182]	1	1	Indonesia	28	No	1998	CQ3	36	Hb	38·9	14 (-)	Not stated	9·5 (-)	Not stated	Not stated	Not stated	Not reported
Maguire-2002 [183]	2	1	Indonesia	28	No	Not stated	CQ3	73	Hb	62·5	32 (-)	Not stated	Not stated	Not stated	Not stated	Not stated	Not reported
Mohapatra-2002 [174]	1	1	India	365	No	1998-2000	CQ3+PQ14	110	Hb	36·4	Not stated	Not stated	8·6 (-)[†]	Not stated	Not stated	Not stated	4 deficient 106 normal
Tjitra-2002 [184]	3	1	Indonesia	28	Yes	1999	CQ3; CQ3+SP; AS+SP	9	Hb	33	8·8 (-)	Not stated	10·7 (-)	Not stated	Not stated	Not stated	Not reported
Krudsood-2006 [173]	2	1	Thailand	28	Yes	2004-2005	CQ3+PQ7; CQ3+EQ	141	Hb and Hct	74·6	25·0 (6·7)	Not stated	Not stated	Not stated	36·6 (-)[†]	Not stated	7 deficient 134 normal
Tasanor-2006 [185]	2	1	Thailand	28	Yes	2002-2004	CQ3+PQ14; Qu+PQ14	31	Type not stated	41·9	Not stated	22	Not stated	Not stated	Not stated	Not stated	31 normal
Kolaczinski-2007 [178]	2	1	Afghanistan	42	Yes	2004	CQ3; SP+AS	96	Hct	43	Not stated	8·5	Not stated	Not stated	35·3 (-)	Not stated	Not reported
Krudsood-2007 [186]	2	1	Thailand	28	Yes	2004-2005	CQ3; AL	51	Hct	31·4	24·3 (6·3)	Not stated	Not stated	Not stated	37·4 (6·1)	Not stated	Not reported
Barnadas-2008 [179]	1	3	Madagascar	28	No	2006	CQ3	105	Hb	53	11·2 (-)	Not stated	10·2 (-)	Not stated	Not stated	Not stated	Not reported
Carmona-Fonseca-2008 [176]	1	2	Colombia	30	Yes	2004	CQ3; CQ3	82	Hb and Hct	62	Not stated	Not stated	10·3 (2·9)	Not stated	31·3 (4·8)	Not stated	Not reported
Lee-2009 [172]	1	1	Republic of Korea	28	No	2007	CQ3+PQ14	142	Hb	0	Not stated	21 (19-50)	13·6 (1·4)	Not stated	Not stated	Not stated	Not reported
Daneshvar-2010 [177]	1	1	Malaysia	28	No	2006-2007	CQ3+PQ14	23	Type not stated	0	38·5 (7·6)	Not stated	Not stated	Not stated	Not stated	Not stated	23 normal
Kinzer-2010 [187]	1	1	Vanuatu	28	No	2005	CQ3	21	Hb	66·7	Not stated	11 (5-53)	Not stated	Not stated	Not stated	Not stated	Not reported
Asih-2011 [188]	1	1	Indonesia	28	No	2007	CQ3	73	Hb	50·7	Not stated	13·2 (2-60)	Not stated	11·8	Not stated	Not stated	Not reported
Maneeboonyang-2011 [189]	2	1	Thailand	90	Yes	2005-2006	CQ3+PQ14; CQ3+PQ14	92	Hct	40	Not stated	Not stated	Not stated	Not stated	38·1 (-)[†]	Not stated	Not reported
Saravu-2012 [170]	1	1	India	28	No	2007-2009	CQ3+PQ14	110	Hb	26·3	Not stated	28·5	Not stated	Not stated	Not stated	Not stated	All tested – results not reported

Ganguly-2013 [190]	2	1	India	42	Yes	2011-2012	CQ3; CQ3+PQ14	250	Hb	10·8	25·2 (-)	Not stated	12·6 (-)	Not stated	Not stated	Not stated	250 normal
Liu-2013 [191]	2	1	China	365	Yes	2009-2010	CQ3+PQ8; ART+NQ	132	Hb	14	Not stated	Not stated	14·6 (1·7)	Not stated	Not stated	Not stated	Not reported
Macareo-2013 [192]	2	1	Thailand	90	Yes	Not stated	CQ5+PQ14; CQ5+TND	6	Type not stated	Not stated	Not stated	Not stated	Not stated	Not stated	Not stated	Not stated	6 normal
Amaratunga-2014 [193]	1	1	Cambodia	28	No	2012-2013	CQ3	87	Hct	20·7	Not stated	26 (4-68)	Not stated	Not stated	Not stated	39	Not reported
Llanos-Cuentas-2014 [55]	6	4	Brazil	180	Yes	2011-2013	CQ3; CQ3+PQ14; CQ3+TQ; CQ3+TQ; CQ3+TQ; CQ3+TQ	104	Hb	28·8	34·8 (-)	Not stated	12·5 (-)	Not stated	Not stated	Not stated	104 normal
Rajgor-2014 [194]		1	India	180	Yes	Not stated	CQ3; CQ3+PQ7; CQ3+PQ14; CQ3+PQ14	1556	Hb	4·8	31·2 (-)	Not stated	Not stated	Not stated	Not stated	Not stated	All tested – results not reported
Shalini-2014 [175]	1	1	India	28	No	2010	CQ3	125	Hb	5	25·9 (10·5)	Not stated	14·4 (1)	Not stated	Not stated	Not stated	Not reported
Assefa-2015 [195]	1	1	Ethiopia	28	No	2014	CQ3	63	Hb	41·7	Not stated	23 (4-59)	11·5 (-)	Not stated	Not stated	Not stated	Not reported
Pareek-2015 [196]	3	8	India	180	Yes	Not stated	CQ3+PQ7; CQ3+PQ14; CQ3+PQ14	358	Hb	17·3	Not stated	20	Not stated	Not stated	Not stated	Not stated	358 normal
Beyene-2016 [197]	1	1	Ethiopia	28	No	2014	CQ3	76	Hb	32	Not stated	19 (3-54)	12·2 (-)	Not stated	Not stated	Not stated	Not reported
Negreiros-2016 [198]	1	1	Brazil	168	No	2014	CQ3+PQ7	119	Hb	45·4	Not stated	23·4 (5-67·3)	Not stated	14·3	Not stated	Not stated	119 normal
Valecha-2016 [171]	2	9	India	42	Yes	2011-2013	CQ3+PQ14; ATM+PIP+PQ14	158	Hct	8·2	33·7 (13·5)	Not stated	Not stated	Not stated	37·8 (5·3)	37·75	158 normal
Seifu-2017 [199]	1	1	Ethiopia	28	No	2013	CQ3	87	Hct	29·7	Not stated	20 (1-65)	Not stated	Not stated	35·5 (-)	Not stated	Not reported

AL – artemether-lumefantrine; Am - artemether; ART - artemisinin; AS – artesunate; ATM – arterolane maleate; Az – azithromycin; BQ – bulaquine; CQ – chloroquine; EQ – elubaquine; Halo – halofantrine; Hb – haemoglobin; Hct – haematocrit; Mfq – mefloquine; NQ - naphthoquine; PIP – piperaquine; PQ – primaquine; Qu – Quinine; SD – standard deviation; SP – sulfadoxine-pyrimethamine; TND – tinidazole; TQ – tafenoquine; * Treatment arms in study described as drug and number of days given if CQ+/-PQ; † Recalculated from subgroups within study.

Table S4. Country of origin and background prevalence of G6PD deficiency in patients with unknown G6PD status (n=1,701)

Country	Number of included patients with unknown G6PD status	Background prevalence of G6PD deficiency (%)*
Afghanistan	265	13
Bhutan	4	10
Bolivia	96	0
Brazil	502	8
Ethiopia	474	2
India	11	14
Indonesia	82	12
Thailand	1	24
Vietnam	266	15

* Percentage with G6PD deficiency was based upon data from Howes *et al*, PLoS Med 2012; 9(11):e100139.

In total 136 (8·0%) of the 1,701 patients would be expected to have G6PD deficiency, assuming equivalent prevalence in patients with *P. vivax* to the background population.

Table S5. Planned primaquine regimens

Total planned dose (mg/kg)	Regimen	Country	Number (%) n=1,446
3·5	0·25 mg/kg/d x 14 days	Indonesia	259 (19·7%)
3·5	0·25 mg/kg/d x 14 days	India	26 (19·7%)
3·5-4·5	0·5 mg/kg/d x 7-9 days	Brazil	313 (28·9%)
5·0	0·5 mg/kg/d x 10 days	Vietnam	260 (18·0%)
7·0	1 mg/kg/d x 7 days	Thailand	170 (11·8%)
7·0	0·5 mg/kg/d x 14 days	Thailand	359 (28·9%)
7·0	0·5 mg/kg/d x 14 days	Pakistan	55 (28·9%)
7·0	0·5 mg/kg/d x 14 days	Malaysia	4 (28·9%)

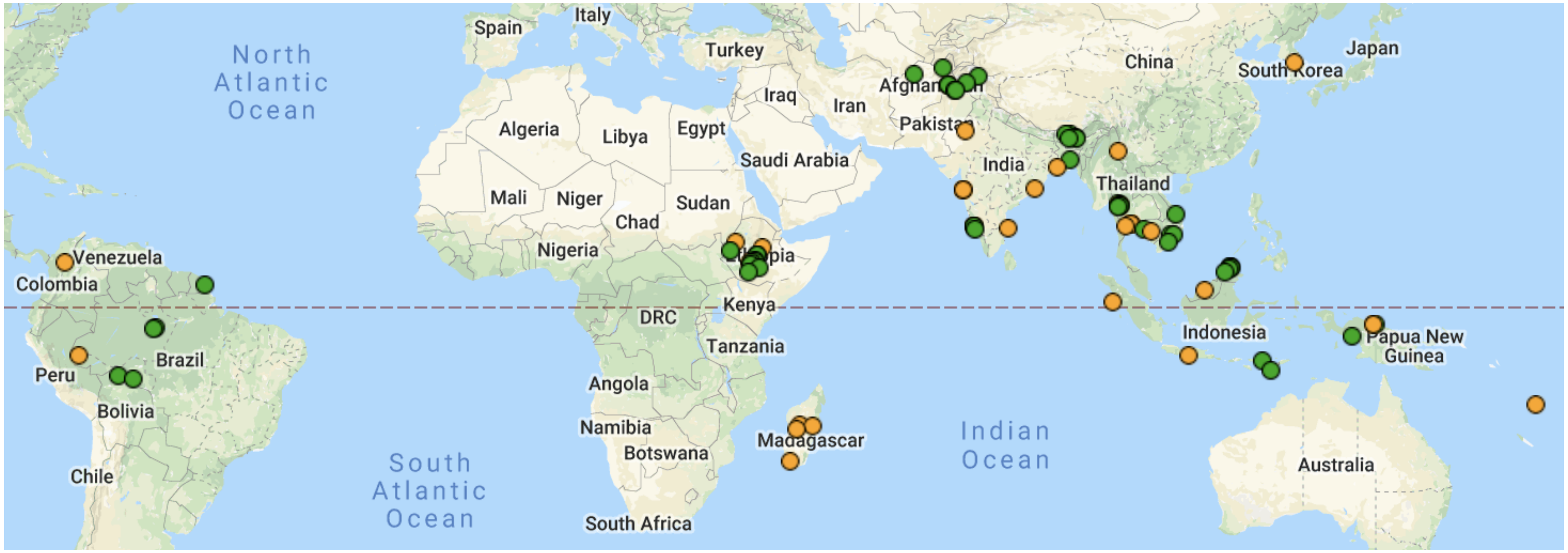

Figure S1. Study sites for clinical trials

Green – included; Orange – targeted but not included.

Table S6. Demographics, baseline characteristics and baseline haemoglobin measurements of G6PD normal patients

	Chloroquine alone			Chloroquine plus primaquine			Overall		
	**Number (%)***	**Mean Hb (SD)**	**Range**	**Number (%)***	**Mean Hb (SD)**	**Range**	**Number (%)***	**Mean Hb (SD)**	**Range**
Overall	856 (100)	12.4 (1.9)	6.5 to 18.1	836 (100)	12.8 (2.0)	5.4 to 19.0	1692 (100)	12.6 (2.0)	5.4 to 19.0
Parasitaemia, parasites per uL; median (IQR)	5280 (1793, 12434)			2688 (945, 8792)			3880 (1280, 10360)		
Gender									
Female	344 (40.2)	11.9 (1.7)	6.5 to 17.3	233 (27.9)	11.7 (1.6)	7.3 to 17.4	577 (34.1)	11.8 (1.7)	6.5 to 17.4
Male	512 (59.8)	12.8 (2.0)	7.0 to 18.1	603 (72.1)	13.3 (2.0)	5.4 to 19.0	1115 (65.9)	13.0 (2.0)	5.4 to 19.0
Age category, years									
<5	56 (6.5)	10.4 (1.5)	7.0 to 14.0	29 (3.5)	10.5 (1.5)	7.3 to 14.1	85 (5.0)	10.4 (1.5)	7.0 to 14.1
5 to <15	331 (38.7)	11.7 (1.6)	7.5 to 16.0	194 (23.2)	11.6 (1.5)	7.5 to 16.3	525 (31.0)	11.7 (1.6)	7.5 to 16.3
≥15	469 (54.8)	13.1 (1.9)	6.5 to 18.1	613 (73.3)	13.3 (1.9)	5.4 to 19.0	1082 (63.9)	13.2 (1.9)	5.4 to 19.0
Weight category, kg									
5 to <15	63 (7.4)	10.4 (1.7)	7.0 to 14.8	31 (3.7)	10.4 (1.3)	7.3 to 13.2	94 (5.6)	10.4 (1.5)	7.0 to 14.8
15 to <25	192 (22.4)	11.5 (1.5)	7.5 to 16.0	97 (11.6)	11.3 (1.4)	8.2 to 15.9	289 (17.1)	11.4 (1.5)	7.5 to 16.0
25 to <35	84 (9.8)	12.1 (1.4)	9.0 to 15.1	62 (7.4)	11.8 (1.5)	7.5 to 15.1	146 (8.6)	12.0 (1.4)	7.5 to 15.1
35 to <45	116 (13.6)	12.2 (1.8)	7.1 to 17.1	90 (10.8)	12.4 (1.8)	6.4 to 17.1	206 (12.2)	12.3 (1.8)	6.4 to 17.1
45 to <55	236 (27.6)	13.1 (1.7)	6.5 to 17.8	244 (29.2)	13.0 (1.9)	5.4 to 17.8	480 (28.4)	13.1 (1.8)	5.4 to 17.8
55 to <80	162 (18.9)	13.6 (1.8)	7.1 to 18.1	285 (34.1)	13.6 (1.9)	5.8 to 19.0	447 (26.4)	13.6 (1.9)	5.8 to 19.0
≥80	3 (0.4)	14.3 (0.2)	14.1 to 14.5	27 (3.2)	14.2 (1.5)	10.5 to 16.8	30 (1.8)	14.2 (1.4)	10.5 to 16.8
Relapse Periodicity									
Long	332 (38.8)	12.7 (1.9)	7.1 to 18.1	303 (36.2)	13.3 (2.0)	5.4 to 18.2	635 (37.5)	13.0 (2.0)	5.4 to 18.2
Short	524 (61.2)	12.2 (1.9)	6.5 to 17.8	533 (63.8)	12.6 (1.9)	7.3 to 19.0	1057 (62.5)	12.4 (1.9)	6.5 to 19.0
Geographical region									
Asia-Pacific	757 (88.4)	12.2 (1.9)	6.5 to 17.8	836 (100)	12.8 (2.0)	5.4 to 19.0	1593 (94.1)	12.5 (2.0)	5.4 to 19.0
The Americas	0 (0)	-	-	0 (0)	-	-	0 (0)	-	-
Africa	99 (11.6)	13.6 (1.7)	10.2 to 18.1	0 (0)	-	-	99 (5.9)	13.6 (1.7)	10.2 to 18.1

Hb – haemoglobin; SD – standard deviation; IQR – interquartile range; * Number of patients (percentage of total patients in group) unless otherwise specified.

Table S7. Demographics, baseline characteristics and baseline haemoglobin measurements of patients with unknown G6PD status

	Chloroquine alone			Chloroquine plus primaquine			Overall		
	**Number (%)***	**Mean Hb (SD)**	**Range**	**Number (%)***	**Mean Hb (SD)**	**Range**	**Number (%)***	**Mean Hb (SD)**	**Range**
Overall	1092 (100)	12.1 (2.2)	6.0 to 18.7	609 (100)	12.5 (2.2)	4.0 to 18.9	1701 (100)	12.2 (2.2)	4.0 to 18.9
Parasitaemia, parasites per uL; median (IQR)	2696 (1118, 6302)			2720 (870, 5720)			2698 (1026, 6120)		
Gender									
Female	422 (38.6)	11.7 (2.0)	6.0 to 17.4	205 (33.7)	11.6 (1.9)	4.0 to 17.2	627 (36.9)	11.7 (2.0)	4.0 to 17.4
Male	670 (61.4)	12.3 (2.2)	6.6 to 18.7	404 (66.3)	12.9 (2.3)	4.9 to 18.9	1074 (63.1)	12.6 (2.2)	4.9 to 18.9
Age category, years									
<5	169 (15.5)	10.8 (2.2)	6.0 to 16.6	43 (7.1)	10.1 (2.0)	4.9 to 13.4	212 (12.5)	10.7 (2.1)	4.9 to 16.6
5 to <15	355 (32.5)	11.5 (2.0)	6.6 to 17.4	132 (21.7)	11.3 (1.8)	5.5 to 14.5	487 (28.6)	11.5 (2.0)	5.5 to 17.4
≥15	568 (52.0)	12.8 (2.0)	6.2 to 18.7	434 (71.3)	13.1 (2.1)	4.0 to 18.9	1002 (58.9)	12.9 (2.0)	4.0 to 18.9
Weight category, kg									
5 to <15	132 (12.1)	10.4 (2.0)	6.0 to 16.3	52 (8.5)	10.2 (1.8)	5.2 to 13.4	184 (10.8)	10.4 (1.9)	5.2 to 16.3
15 to <25	244 (22.3)	11.6 (2.1)	6.9 to 16.6	75 (12.3)	10.9 (1.9)	4.9 to 14.5	319 (18.8)	11.4 (2.1)	4.9 to 16.6
25 to <35	98 (9.0)	11.4 (1.8)	6.6 to 16.2	32 (5.3)	11.4 (1.9)	7.8 to 14.6	130 (7.6)	11.4 (1.8)	6.6 to 16.2
35 to <45	79 (7.2)	11.8 (1.9)	6.5 to 17.4	63 (10.3)	11.7 (2.1)	5.8 to 15.3	142 (8.3)	11.8 (2.0)	5.8 to 17.4
45 to <55	157 (14.4)	12.5 (2.2)	6.2 to 18.7	93 (15.3)	12.6 (2.1)	6.0 to 18.1	250 (14.7)	12.6 (2.1)	6.0 to 18.7
55 to <80	311 (28.5)	12.9 (1.9)	7.0 to 18.0	223 (36.6)	13.5 (1.8)	4.0 to 18.9	534 (31.4)	13.1 (1.9)	4.0 to 18.9
≥80	71 (6.5)	13.8 (1.4)	9.9 to 16.5	71 (11.7)	13.9 (1.7)	8.2 to 17.9	142 (8.3)	13.8 (1.6)	8.2 to 17.9
Relapse Periodicity									
Long	1024 (93.8)	12.1 (2.1)	6.0 to 18.0	324 (53.2)	13.5 (1.8)	4.0 to 18.9	1348 (79.2)	12.4 (2.2)	4.0 to 18.9
Short	68 (6.2)	11.7 (2.4)	6.2 to 18.7	285 (46.8)	11.4 (2.2)	4.9 to 18.1	353 (20.8)	11.5 (2.2)	4.9 to 18.7
Geographical region									
Asia-Pacific	333 (30.5)	11.1 (1.7)	6.2 to 18.7	296 (48.6)	11.5 (2.2)	4.9 to 18.1	629 (37.0)	11.3 (2.0)	4.9 to 18.7
The Americas	285 (26.1)	12.5 (2.0)	7.0 to 17.4	313 (51.4)	13.5 (1.8)	4.0 to 18.9	598 (35.2)	13.0 (2.0)	4.0 to 18.9
Africa	474 (43.4)	12.6 (2.3)	6.0 to 18.0	0 (0)	-	-	474 (27.9)	12.6 (2.3)	6.0 to 18.0

Hb – haemoglobin; SD – standard deviation; IQR – interquartile range; * Number of patients (percentage of total patients in group) unless otherwise specified.

Table S8. Comparison of baseline characteristics between included and targeted studies

Characteristic	Included studies (n=29)	Targeted studies* (n=31)
Region		
Asia-Pacific, studies (%)	20 (69·0%)	24 (77·4%)
Africa, studies (%)	4 (13·8%)	4 (12·9%)
The Americas, studies (%)	5 (17·2%)	3 (9·7%)
Year of enrolment†		
Pre-2009, studies (%)	9 (31·0%)	16 (59·3%)‡
2009-2017, studies (%)	20 (69·0%)	11 (40·7%)‡
Age, mean (SD)	22·1 (15·1)	28·3 (4·9)§
Female, % of patients, mean (SD)	35·4%	22·2 (19·5)¶
Baseline Hb, mean (SD)	12·4 (2·1)	12·0 (1·3)‖

SD – standard deviation; Hb – haemoglobin; * Age, female percentage and baseline haemoglobin of targeted studies calculated using frequency weighted mean according to number of patients treated with chloroquine alone or chloroquine and primaquine; † Year of enrolment defined as the year study enrolment completed; ‡ Year of enrolment not available from four studies; § Mean age not available from 17 studies; ¶ Percentage not available from one study; || Percentage not available from 11 studies and haemoglobin recalculated from haematocrit for eight studies.

Table S9. Risk factors for baseline anaemia (Hb < 10 g/dL)

	Number with anaemia (%) n=3,421	Unadjusted Odds Ratio (95% CI)	p value	Adjusted Odds Ratio (95% CI)	p value
Gender					
Male	205/2211 (9·3%)	Reference	-	Reference	-
Female	180/1210 (14·9%)	1·71 (1·27, 2·29)	0·0003	1·34 (1·05, 1·71)	0·0188
Age category, years					
≥15	118/2107 (5·6%)	Reference	-	Reference	-
<5	114/297 (38·4%)	10·50 (5·86, 18·82)	<0·0001	10·37 (6·09, 17·67)	<0·0001
5 to <15	153/1017 (15·0%)	2·98 (2·07, 4·31)	<0·0001	3·07 (2·07, 4·54)	<0·0001
G6PD status					
Normal	149/1692 (8·8%)	Reference	-	Reference	-
Borderline	0/3 (0·0%)	-	-	-	-
Deficient	4/25 (16·0%)	1·97 (0·79, 4·94)	0·1466	2·88 (1·14, 7·32)	0·0259
Not known	232/1701 (13·6%)	1·64 (0·88, 3·03)	0·1174	1·59 (0·93, 2·73)	0·0897
Relapse periodicity					
Long	199/1987 (10·0%)	Reference	-	Reference	-
Short	186/1434 (13·0%)	1·34 (0·69, 2·60)	0·3889	1·94 (1·01, 3·71)	0·0470
Geographical region					
Asia-Pacific	275/2247 (12·2%)	Reference	-	-	-
Africa	65/598 (11·3%)	0·91 (0·37, 2·25)	0·8416	-	-
The Americas	45/576 (7·5%)	0·58 (0·16, 2·19)	0·4247	-	-
Parasitaemia, parasites per uL every ten times increase	-	1·20 (0·84, 1·70)	0·3244	0·96 (0·72, 1·28)	0·7738
*Temperature >37·5°C at baseline**					
Absent	179/1808 (9·9%)	Reference	-	Reference	-
Present	150/1280 (11·7%)	1·21 (0·83, 1·75)	0·3166	1·10 (0·76, 1·61)	0·6100

Covariates to include in the model were decided *a priori*. Geographical region was not included in the multivariable model due to collinearity with relapse periodicity. * Data on baseline temperature was only available for 3,088 patients.

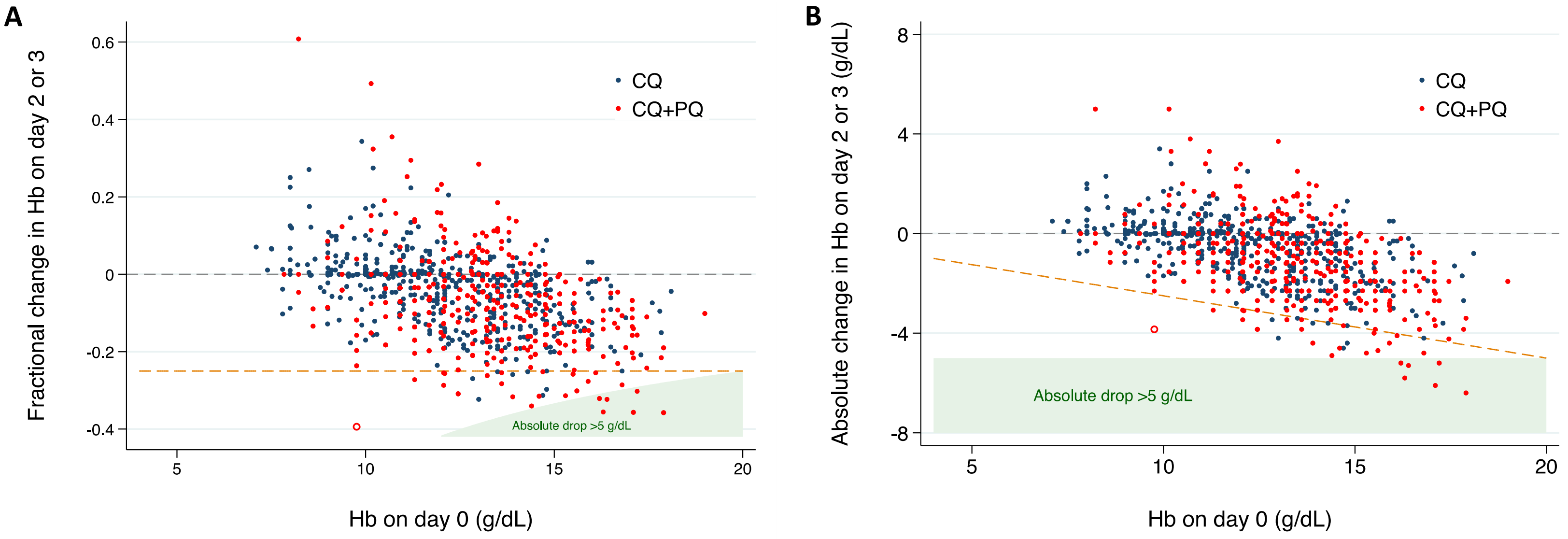

Figure S2. Relationship between day 0 haemoglobin and haemoglobin on day 2/3 in patients treated with chloroquine alone (n=610) and chloroquine plus primaquine (n=471) as (A) fractional change or (B) absolute change.

Open circle represents the single patient with a clinically significant fall >25% to <7 g/dL at day 2/3 (male patient with normal G6PD status). Dashed orange line represents a fractional fall of 25%. The fitted linear regression model estimated the relationship in patients with CQ alone to be: Absolute change in Hb on day 2/3 = (Day 0 Hb – 11·55)/-1·04. The baseline Hb correlates negatively with the fractional change in Hb at day 2/3 (r=-0·463 [95%CI -0·509 to -0·415], p<0·0001).

Table S10. Sensitivity analysis for estimated mean change in haemoglobin for patients treated with chloroquine and primaquine compared to chloroquine (reference group)

Timepoint	Range of estimated mean Hb change g/dL	Coefficient of Variation (%)*
Day of nadir	-0·23 to 0†	23·53
Day 7	-0·45 to -0·26	7·44
Day 42	0·33 to 0·65	9·19

Sensitivity analysis was generated by removing each study site one at a time

There were a total of 47 sites; * The coefficient of variation calculated as standard deviation divided by the mean of the estimates; † Removal of 45 of the 47 study sites had an estimated mean haemoglobin (Hb) change of -0·19 to -0·08 g/dL at nadir. Removal of patients from Jalalabad from Awab *et al* [29] led to an estimated mean Hb change of 0 g/dL at nadir and removal of Thanh *et al* [40] led to estimated change of -0·23 g/dL.

Table S11. Factors associated with change in haemoglobin between day 0 and day 2/3 in G6PD normal patients

	Number of patients n=672	Crude change in haemoglobin*, g/dL (95% CI)	p value	Adjusted change in haemoglobin, g/dL (95% CI)	p value
Baseline haemoglobin, g/dL every gram increase	672	-0.28 (-0.32, -0.24)	<0·0001	-0.37 (-0.42, -0.32)	<0·0001
Gender					
Male	417	Reference	-	Reference	-
Female	255	-0.34 (-0.52, -0.16)	0·0002	-0.37 (-0.54, -0.19)	<0·0001
Age category, years					
≥15	19	Reference	-	Reference	-
<5	251	-0.96 (-1.47, -0.44)	0·0003	-0.93 (-1.43, -0.42)	0·0003
5 to <15	402	-0.52 (-0.72, -0.32)	<0·0001	-0.58 (-0.78, -0.38)	<0·0001
Primaquine use					
No	338	Reference	-	Reference	-
Yes	334	-0.18 (-0.59, 0.22)	0·3773	-0.14 (-0.41, 0.14)	0·3296
Relapse periodicity					
Long	260	Reference	-	Reference	-
Short	412	-0.12 (-0.44, 0.21)	0·4808	-0.33 (-0.62, -0.04)	0·0259
Chloroquine dose, mg/kg, every mg/kg increase	672	-0.04 (-0.07, -0.004)	0·0276	-0.02 (-0.06, 0.01)	0·1838
Parasitaemia, parasites per uL every ten times increase	672	-0.31 (-0.44, -0.17)	<0·0001	-0.33 (-0.46, -0.20)	<0·0001

Analysis undertaken in 672 patients with haemoglobin data available for day 2/3; * Adjusted for baseline haemoglobin

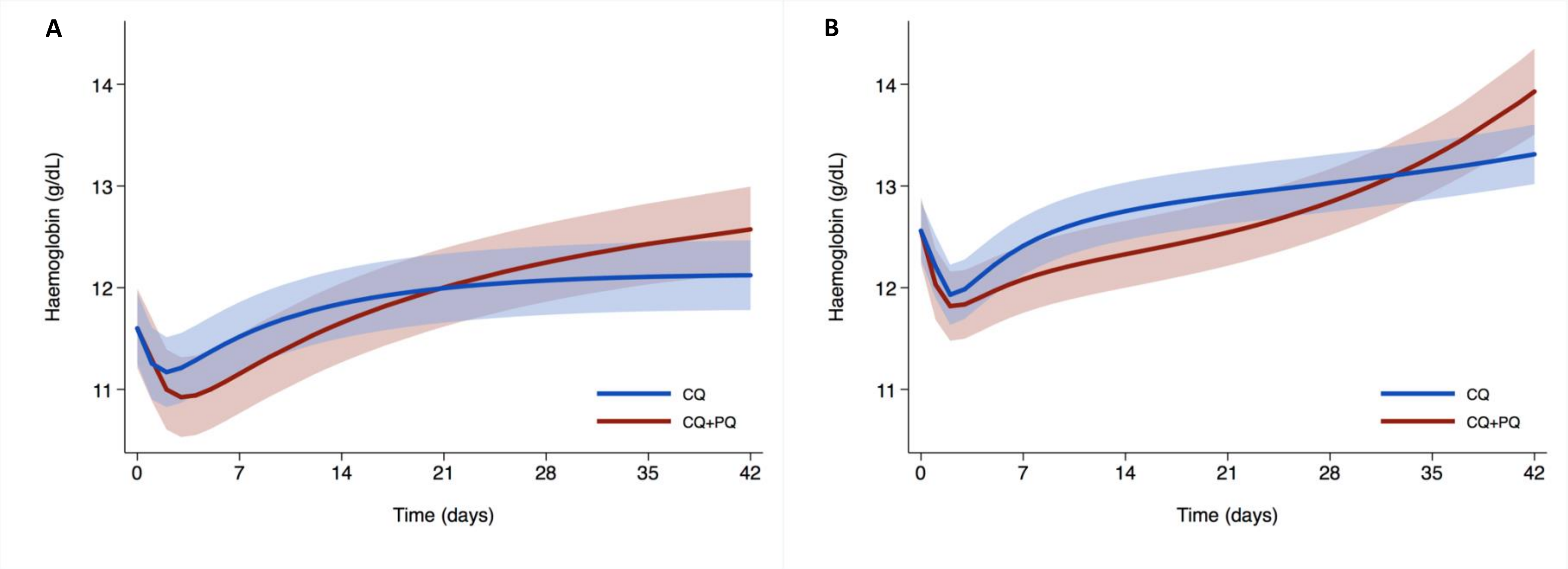

Figure S3. Mean haemoglobin versus time profiles for (a) female patients treated with chloroquine with (n=438) or without (n=772) primaquine and (b) male patients treated with chloroquine with (n=1,008) or without (n=1,203) primaquine.

CQ – chloroquine; PQ – primaquine. Profiles for chloroquine alone and chloroquine plus primaquine adjusted to the same baseline haemoglobin. Figures derived from linear mixed effect models with fractional polynomial terms for time. Shaded regions show 95% confidence intervals.

Table S12: Patients with a Hb fall >25% leading to development of severe anaemia (Hb <7 g/dL) during the first 42 days

	Age (yrs)	Gender	Study	Country	Periodicity	CQ total dose (mg/kg)	G6PD status	Primaquine	PQ total dose (mg/kg)	PQ regimen	Recurrence	Day0 Hb (g/dL)	Minimum Hb (g/dL)	Absolute Hb drop (g/dL)	Percentage Hb drop	Day of minimum
1	1·5	M	Getachew-2015	Ethiopia	Long	44·12	Unknown	No	-	None	No	11·0	6·6	4·4	40·0%	28
2	3	F	Thanh-2015	Vietnam	Short	22·50	Unknown	Yes	3·75	10 days from day 0	No	10·5	6·6	3·9	37·1%	28
3	5	F	Thanh-2015	Vietnam	Short	30·00	Unknown	Yes	5·00	10 days from day 0	No	13·3	6·5	6·8	51·1%	28
4	5	M	Chu-2018a	Thailand	Short	29·87	Normal	Yes	7·11	7 days from day 0	No	9·8	5·9	3·8	39·4%	3
5	8	M	Thanh-2015	Vietnam	Short	26·47	Unknown	Yes	4·41	10 days from day 0	No	11·0	4·6	6·4	58·2%	14
6	9	M	Thanh-2015	Vietnam	Short	27·27	Unknown	Yes	5·11	10 days from day 0	No	13·1	5·9	7·2	55·0%	14
7	26	M	Thanh-2015	Vietnam	Short	28·30	Deficient	Yes	4·60	10 days from day 0	No	14·0	6·6	7·4	52·9%	14
8	30	F	Chu-2018a	Thailand	Short	23·67	Normal	Yes	7·68	7 days from day 0	No	13·2	5·9	7·3	55·3%	7
9	40	F	Thanh-2015	Vietnam	Short	29·27	Unknown	Yes	5·03	10 days from day 0	No	8·3	5·0	3·3	39·8%	14

Table S13. Patients with haemoglobin falling >5 g/dL during the first 42 days

	Age (yrs)	Gender	Study	Country	Periodicity	CQ total dose (mg/kg)	G6PD status	PQ	PQ total dose (mg/kg)	PQ regimen	Recurrence	Day0 Hb (g/dL)	Minimum Hb (g/dL)	Absolute Hb drop (g/dL)	Percentage Hb drop	Day of minimum
1	25	F	Leslie-2007	Pakistan	Long	26·16	Normal	No	-	None	No	16·5	11·1	5·4	32·7%	1
2	28	M	Getachew-2015	Ethiopia	Long	29·59	Unknown	No	-	None	No	14·9	8·0	6·9	46·3%	28
3	38	M	Phyo-2011	Thailand	Short	23·86	Normal	No	-	None	No	16·3	9·8	6·5	40·1%	23
4	41·3	M	Taylor-2001	Indonesia	Short	34·62	Unknown	No	-	None	Yes	13·8	8·2	5·6	40·6%	7
5	5	F	Thanh-2015	Vietnam	Short	30·00	Unknown	Yes	5·00	10 days	No	13·3	6·5	6·8	51·1%	28
6	8	M	Thanh-2015	Vietnam	Short	26·47	Unknown	Yes	4·41	10 days	No	11·0	4·6	6·4	58·2%	14
7	9	M	Thanh-2015	Vietnam	Short	27·27	Unknown	Yes	5·11	10 days	No	13·1	5·9	7·2	55·0%	14
8	10	F	Chu-2018a	Thailand	Short	26·78	Normal	Yes	7·24	7 days from day 0	No	13·2	7·5	5·8	43·6%	5
9	14	M	Chu-2018a	Thailand	Short	25·36	Normal	Yes	7·50	14 days from day 0	No	16·3	9·4	6·9	42·5%	8
10	15	M	Chu-2018a	Thailand	Short	25·36	Normal	Yes	7·50	14 days from day 0	No	14·4	8·6	5·8	40·1%	6
11	17	M	Marques-2014	Brazil	Long	23·08	Unknown	Yes	3·23	7 to 9 days	No	16·3	10·5	5·8	35·6%	3
12	18	M	Marques-2014	Brazil	Long	24·19	Unknown	Yes	3·39	7 to 9 days	No	16·2	11·0	5·2	32·1%	3
13	21	M	Thanh-2015	Vietnam	Short	25·00	Unknown	Yes	4·69	10 days	No	16·8	11·1	5·7	33·9%	28
14	22	M	Thanh-2015	Vietnam	Short	27·27	Unknown	Yes	4·69	10 days	No	15·3	10·0	5·3	34·6%	14
15	23	F	Marques-2014	Brazil	Long	29·41	Unknown	Yes	4·12	7 to 9 days	No	17·2	12·0	5·2	30·2%	3
16	25	M	Marques-2014	Brazil	Long	21·74	Unknown	Yes	3·04	7 to 9 days	No	17·1	11·0	6·1	35·7%	3
17	25	F	Chu-2018a	Thailand	Short	24·40	Normal	Yes	7·50	14 days from day 0	No	12·8	7·8	5·0	38·9%	4
18	26	M	Thanh-2015	Vietnam	Short	28·30	Deficient	Yes	4·60	10 days	No	14·0	6·6	7·4	52·9%	14
19	26	M	Thanh-2015	Vietnam	Short	25·00	Unknown	Yes	4·69	10 days	No	15·7	8·8	6·9	43·9%	14
20	28	M	Chu-2018a	Thailand	Short	24·85	Normal	Yes	7·35	7 days from day 0	No	14·4	7·1	7·3	50·8%	4
21	30	M	Marques-2014	Brazil	Long	19·74	Unknown	Yes	2·96	7 to 9 days	No	17·9	11·5	6·4	35·8%	3
22	30	F	Chu-2018a	Thailand	Short	23·67	Normal	Yes	7·68	7 days from day 0	No	13·2	5·9	7·3	55·3%	7
23	40	M	Marques-2014	Brazil	Long	23·44	Unknown	Yes	3·28	7 to 9 days	Yes	16·4	11·1	5·3	32·3%	3

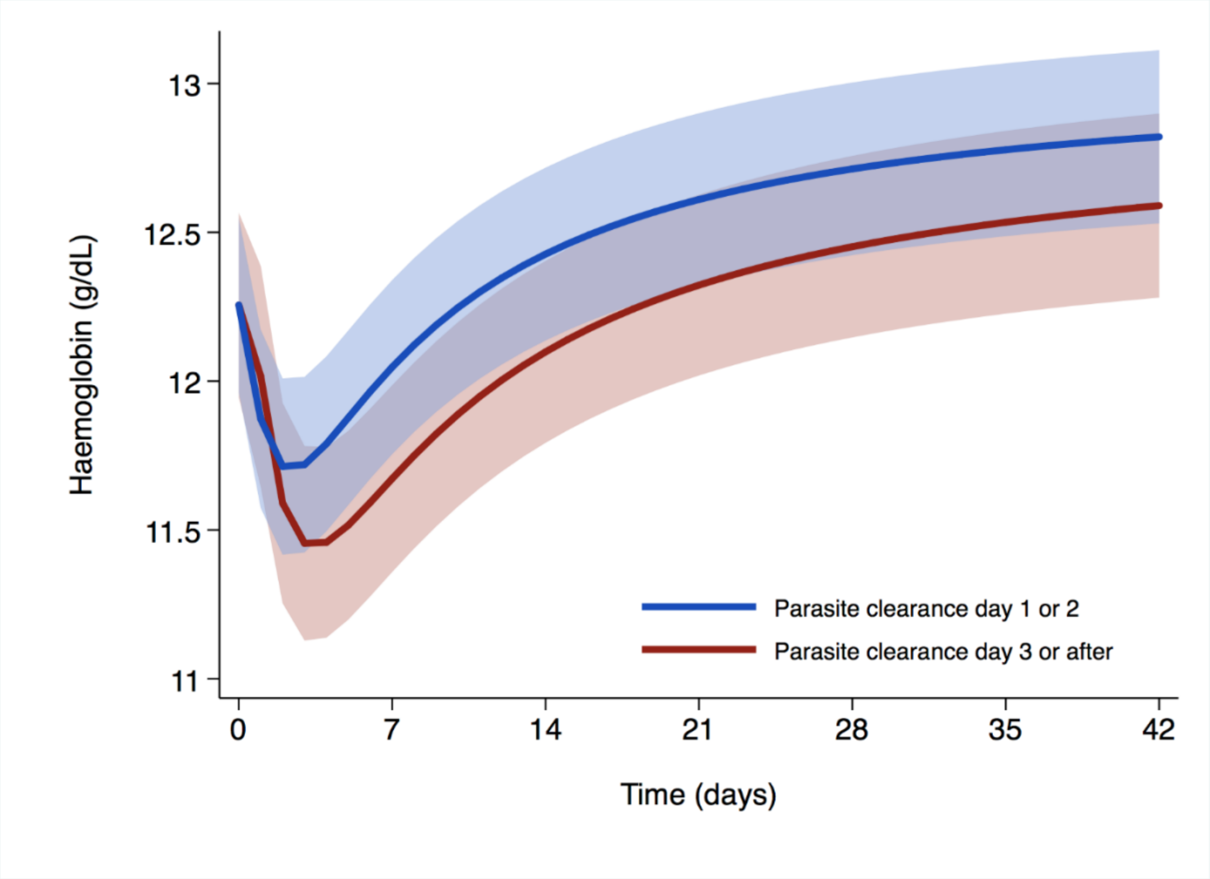

Figure S4. Mean haemoglobin versus time profile for patients with (n=622) or without (n=2,076) delayed parasite clearance.

Profiles adjusted to the same baseline haemoglobin. Figures derived from linear mixed effect models with fractional polynomial terms for time. Shaded regions show 95% confidence intervals.

Table S14. Unadjusted absolute and percentage change in haemoglobin and risk of anaemia if G6PD deficient

Day and metric	Chloroquine alone	Chloroquine plus primaquine
Day 2/3 (number of patients)	14	0
Absolute change*, mean (SD) [range]; g/dL	-0·7 (1·1) [-2·3 to 1·2]	-
Percentage change*, mean (SD) [range]; %	-5·5 (8·1) [-17·6 to 10·7]	-
Percentage fall >25%	0/14 (0%)	-
>25% fall associated with severe anaemia (%)†	0/14 (0%)	-
Absolute fall >5 g/dL‡	0/14 (0%)	-
Day 7 ±2	26	0
Absolute change*, mean (SD) [range]; g/dL	-0·3 (1·3) [-2·7 to 1·9]	-
Percentage change*, mean (SD) [range]; %	-1·8 (10·2) [-18·6 to 16·4]	-
Percentage fall >25%	0/26 (0%)	-
>25% fall associated with severe anaemia (%)†	0/26 (0%)	-
Absolute fall >5 g/dL‡	0/26 (0%)	-
Day 28 ±3	22	1
Absolute change*, mean (SD) [range]; g/dL	0·4 (1·1) [-2·5 to 2·7]	-0·4 (-)
Percentage change*, mean (SD) [range]; %	4·1 (9·7) [-17·2 to 31·3]	-2·9 (-)
Percentage fall >25%	0/22 (0%)	0/1 (0%)
>25% fall associated with severe anaemia (%)†	0/22 (0%)	0/1 (0%)
Absolute fall >5 g/dL‡	0/22 (0%)	0/1 (0%)

SD – standard deviation; * Results are reported as a change in haemoglobin (Hb), with positive results reflecting a rise in Hb and negative results reflecting a fall in Hb; † Patients were considered to develop severe anaemia if their baseline Hb was ≥7 g/dL and their follow up Hb was <7 g/dL, with the denominator the number of people with a Hb recorded for that day who had a baseline ≥7 g/dL. All patients that developed severe anaemia had a Hb fall >25%. Table S12 provides additional patient details; ‡ Table S13 provides additional patient details.

References S1. Studies not included in analysis

55. Llanos-Cuentas A, Lacerda MV, Rueangweerayut R, et al. Tafenoquine plus chloroquine for the treatment and relapse prevention of *Plasmodium vivax* malaria (DETECTIVE): a multicentre, double-blind, randomised, phase 2b dose-selection study. *Lancet* 2014; **383**(9922): 1049-58.

58. Pukrittayakamee S, Clemens R, Chantra A, et al. Therapeutic responses to antibacterial drugs in vivax malaria. *Trans R Soc Trop Med Hyg* 2001; **95**(5): 524-8.

59. Lacy MD, Maguire JD, Barcus MJ, et al. Atovaquone/proguanil therapy for *Plasmodium falciparum* and *Plasmodium vivax* malaria in Indonesians who lack clinical immunity. *Clin Infect Dis* 2002; **35**(9): e92-5.

60. Silachamroon U, Krudsood S, Treeprasertsuk S, et al. Clinical trial of oral artesunate with or without high-dose primaquine for the treatment of vivax malaria in Thailand. *Am J Trop Med Hyg* 2003; **69**(1): 14-8.

61. Hamedi Y, Safa O, Zare S, Tan-ariya P, Kojima S, Looareesuwan S. Therapeutic efficacy of artesunate in *Plasmodium vivax* malaria in Thailand. *Southeast Asian J Trop Med Public Health* 2004; **35**(3): 570-4.

62. Khan MZ, Isani Z, Ahmed TM, et al. Efficacy and safety of halofantrine in Pakistani children and adults with malaria caused by *P. falciparum* and *P. vivax*. *Southeast Asian J Trop Med Public Health* 2006; **37**(4): 613-8.

63. Dao NV, Cuong BT, Ngoa ND, et al. Vivax malaria: preliminary observations following a shorter course of treatment with artesunate plus primaquine. *Trans R Soc Trop Med Hyg* 2007; **101**(6): 534-9.

64. Hasugian AR, Purba HL, Kenangalem E, et al. Dihydroartemisinin-piperaquine versus artesunate-amodiaquine: superior efficacy and posttreatment prophylaxis against multidrug-resistant *Plasmodium falciparum* and *Plasmodium vivax* malaria. *Clin Infect Dis* 2007; **44**(8): 1067-74.

65. Ratcliff A, Siswantoro H, Kenangalem E, et al. Two fixed-dose artemisinin combinations for drug-resistant falciparum and vivax malaria in Papua, Indonesia: an open-label randomised comparison. *Lancet* 2007; **369**(9563): 757-65.

66. Barnadas C, Tichit M, Bouchier C, et al. *Plasmodium vivax dhfr* and *dhps* mutations in isolates from Madagascar and therapeutic response to sulphadoxine-pyrimethamine. *Malar J* 2008; **7**: 35.

67. Krudsood S, Tangpukdee N, Wilairatana P, et al. High-dose primaquine regimens against relapse of *Plasmodium vivax* malaria. *Am J Trop Med Hyg* 2008; **78**(5): 736-40.

68. Hasugian AR, Tjitra E, Ratcliff A, et al. *In vivo* and *in vitro* efficacy of amodiaquine monotherapy for treatment of infection by chloroquine-resistant *Plasmodium vivax*. *Antimicrob Agents Chemother* 2009; **53**(3): 1094-9.

69. Congpuong K, Bualombai P, Banmairuroi V, Na-Bangchang K. Compliance with a three-day course of artesunate-mefloquine combination and baseline anti-malarial treatment in an area of Thailand with highly multidrug resistant falciparum malaria. *Malar J* 2010; **9**: 43.

70. Pukrittayakamee S, Imwong M, Chotivanich K, Singhasivanon P, Day NP, White NJ. A comparison of two short-course primaquine regimens for the treatment and radical cure of *Plasmodium vivax* malaria in Thailand. *Am J Trop Med Hyg* 2010; **82**(4): 542-7.

71. Abdallah TM, Ali AA, Bakri M, Gasim GI, Musa IR, Adam I. Efficacy of artemether-lumefantrine as a treatment for uncomplicated *Plasmodium vivax* malaria in eastern Sudan. *Malar J* 2012; **11**: 404.

72. Betuela I, Rosanas-Urgell A, Kiniboro B, et al. Relapses contribute significantly to the risk of *Plasmodium vivax* infection and disease in Papua New Guinean children 1-5 years of age. *J Infect Dis* 2012; **206**(11): 1771-80.

73. Eibach D, Ceron N, Krishnalall K, et al. Therapeutic efficacy of artemether-lumefantrine for *Plasmodium vivax* infections in a prospective study in Guyana. *Malar J* 2012; **11**: 347.

74. Tjitra E, Hasugian AR, Siswantoro H, et al. Efficacy and safety of artemisinin-naphthoquine versus dihydroartemisinin-piperaquine in adult patients with uncomplicated malaria: a multi-centre study in Indonesia. *Malar J* 2012; **11**: 153.

75. Pasaribu AP, Chokejindachai W, Sirivichayakul C, et al. A randomized comparison of dihydroartemisinin-piperaquine and artesunate-amodiaquine combined with primaquine for radical treatment of vivax malaria in Sumatera, Indonesia. *J Infect Dis* 2013; **208**(11): 1906-13.

76. Senn N, Rarau P, Manong D, et al. Effectiveness of artemether/lumefantrine for the treatment of uncomplicated *Plasmodium vivax* and *P. falciparum* malaria in young children in Papua New Guinea. *Clin Infect Dis* 2013; **56**(10): 1413-20.

77. Sutanto I, Tjahjono B, Basri H, et al. Randomized, open-label trial of primaquine against vivax malaria relapse in Indonesia. *Antimicrob Agents Chemother* 2013; **57**(3): 1128-35.

78. Laman M, Moore BR, Benjamin JM, et al. Artemisinin-naphthoquine versus artemether-lumefantrine for uncomplicated malaria in Papua New Guinean children: an open-label randomized trial. *PLoS Med* 2014; **11**(12): e1001773.

79. Lon C, Manning JE, Vanachayangkul P, et al. Efficacy of two versus three-day regimens of dihydroartemisinin-piperaquine for uncomplicated malaria in military personnel in northern Cambodia: an open-label randomized trial. *PLoS One* 2014; **9**(3): e93138.

80. Kheng S, Muth S, Taylor WR, et al. Tolerability and safety of weekly primaquine against relapse of *Plasmodium vivax* in Cambodians with glucose-6-phosphate dehydrogenase deficiency. *BMC Med* 2015; **13**: 203.

81. Nelwan EJ, Ekawati LL, Tjahjono B, et al. Randomized trial of primaquine hypnozoitocidal efficacy when administered with artemisinin-combined blood schizontocides for radical cure of *Plasmodium vivax* in Indonesia. *BMC Med* 2015; **13**: 294.

82. Alecrim MG, Carvalho LM, Fernandes MC, et al. [Malaria treatment with artesunate (retocaps) in children of the Brazilian Amazon]. *Rev Soc Bras Med Trop* 2000; **33**(2): 163-8.

83. Fernandopulle BM, Weeraratne CL, Weerasuriya K, Karunaweera ND. Efficacy of a five-day course of primaquine in preventing relapses in *Plasmodium vivax* malaria--a pilot study. *Ceylon Med J* 2003; **48**(1): 32.

84. Yeramian P, Meshnick SR, Krudsood S, et al. Efficacy of DB289 in Thai patients with *Plasmodium vivax* or acute, uncomplicated *Plasmodium falciparum* infections. *J Infect Dis* 2005; **192**(2): 319-22.

85. Benjamin J, Moore B, Lee ST, et al. Artemisinin-naphthoquine combination therapy for uncomplicated pediatric malaria: a tolerability, safety, and preliminary efficacy study. *Antimicrob Agents Chemother* 2012; **56**(5): 2465-71.

86. Marfurt J, Mueller I, Sie A, et al. Low efficacy of amodiaquine or chloroquine plus sulfadoxine-pyrimethamine against *Plasmodium falciparum* and *P. vivax* malaria in Papua New Guinea. *Am J Trop Med Hyg* 2007; **77**(5): 947-54.

87. Karunajeewa HA, Mueller I, Senn M, et al. A trial of combination antimalarial therapies in children from Papua New Guinea. *N Engl J Med* 2008; **359**(24): 2545-57.

88. Phyo AP, Jittamala P, Nosten FH, et al. Antimalarial activity of artefenomel (OZ439), a novel synthetic antimalarial endoperoxide, in patients with *Plasmodium falciparum* and *Plasmodium vivax* malaria: an open-label phase 2 trial. *Lancet Infect Dis* 2016; **16**(1): 61-9.

89. White NJ, Duong TT, Uthaisin C, et al. Antimalarial Activity of KAF156 in Falciparum and Vivax Malaria. *N Engl J Med* 2016; **375**(12): 1152-60.

90. McGready R, Thwai KL, Cho T, et al. The effects of quinine and chloroquine antimalarial treatments in the first trimester of pregnancy. *Trans R Soc Trop Med Hyg* 2002; **96**(2): 180-4.

91. Moore BR, Benjamin JM, Auyeung SO, et al. Safety, tolerability and pharmacokinetic properties of coadministered azithromycin and piperaquine in pregnant Papua New Guinean women. *Br J Clin Pharmacol* 2016; **82**(1): 199-212.

92. Bergonzoli G, Rivers Cuadra JC. [Therapeutic efficacy of different antimalarial regimens in the Costa Rica-Nicaragua border region]. *Rev Panam Salud Publica* 2000; **7**(6): 366-70.

93. Singh RK. Emergence of chloroquine-resistant vivax malaria in south Bihar (India). *Trans R Soc Trop Med Hyg* 2000; **94**(3): 327.

94. Taylor WR, Doan HN, Nguyen DT, et al. Assessing drug sensitivity of *Plasmodium vivax* to halofantrine or choroquine in southern, central Vietnam using an extended 28-day *in vivo* test and polymerase chain reaction genotyping. *Am J Trop Med Hyg* 2000; **62**(6): 693-7.

95. Villalobos-Salcedo JM, Tada MS, Kimura E, Menezes MJ, Pereira da Silva LH. *In-vivo* sensitivity of *Plasmodium vivax* isolates from Rondonia (western Amazon region, Brazil) to regimens including chloroquine and primaquine. *Ann Trop Med Parasitol* 2000; **94**(8): 749-58.

96. Abdon NP, Pinto AY, das Silva Rdo S, de Souza JM. [Assessment of the response to reduced treatment schemes for vivax malaria]. *Rev Soc Bras Med Trop* 2001; **34**(4): 343-8.

97. Adak T, Valecha N, Sharma VP. *Plasmodium vivax* polymorphism in a clinical drug trial. *Clin Diagn Lab Immunol* 2001; **8**(5): 891-4.

98. Dua VK, Sharma VP. *Plasmodium vivax* relapses after 5 days of primaquine treatment, in some industrial complexes of India. *Ann Trop Med Parasitol* 2001; **95**(7): 655-9.

99. Duarte EC, Pang LW, Ribeiro LC, Fontes CJ. Association of subtherapeutic dosages of a standard drug regimen with failures in preventing relapses of vivax malaria. *Am J Trop Med Hyg* 2001; **65**(5): 471-6.

100. Soto J, Toledo J, Gutierrez P, et al. *Plasmodium vivax* clinically resistant to chloroquine in Colombia. *Am J Trop Med Hyg* 2001; **65**(2): 90-3.

101. Baird JK, Tiwari T, Martin GJ, et al. Chloroquine for the treatment of uncomplicated malaria in Guyana. *Ann Trop Med Parasitol* 2002; **96**(4): 339-48.

102. Castillo CM, Osorio LE, Palma GI. Assessment of therapeutic response of *Plasmodium vivax* and *Plasmodium falciparum* to chloroquine in a Malaria transmission free area in Colombia. *Mem Inst Oswaldo Cruz* 2002; **97**(4): 559-62.

103. Congpuong K, Na-Bangchang K, Thimasarn K, Tasanor U, Wernsdorfer WH. Sensitivity of *Plasmodium vivax* to chloroquine in Sa Kaeo Province, Thailand. *Acta Trop* 2002; **83**(2): 117-21.

104. Hamedi Y, Nateghpour M, Tan-ariya P, Tiensuwan M, Silachamroon U, Looareesuwan S. *Plasmodium vivax* malaria in Southeast Iran in 1999-2001: establishing the response to chloroquine *in vitro* and *in vivo*. *Southeast Asian J Trop Med Public Health* 2002; **33**(3): 512-8.

105. Blair-Trujillo S, Castano AT, Restrepo ME, Sanchez GA, Carmona-Fonseca J. [Adequate clinical and parasitological *Plasmodium vivax* response to chloroquine in Colombia (Turbo, Antioquia), 2001]. *Infectio* 2002; **6**(1): 21-6.

106. Yadav RS, Ghosh SK. Radical curative efficacy of five-day regimen of primaquine for treatment of *Plasmodium vivax* malaria in India. *J Parasitol* 2002; **88**(5): 1042-4.

107. da Silva Rdo S, Pinto AY, Calvosa VS, de Souza JM. [Short course schemes for vivax malaria treatment]. *Rev Soc Bras Med Trop* 2003; **36**(2): 235-9.

108. Machado RL, de Figuereido Filho AF, Calvosa VS, Figueredo MC, Nascimento JM, Povoa MM. Correlation between *Plasmodium vivax* variants in Belem, Para State, Brazil and symptoms and clearance of parasitaemia. *Braz J Infect Dis* 2003; **7**(3): 175-7.

109. Nandy A, Addy M, Maji AK, Bandyopadhyay AK. Monitoring the chloroquine sensitivity of *Plasmodium vivax* from Calcutta and Orissa, India. *Ann Trop Med Parasitol* 2003; **97**(3): 215-20.

110. Pinto AY, Azevedo CH, da Silva JB, de Souza JM. Assessment of chloroquine single dose treatment of malaria due to *Plasmodium vivax* in Brazilian Amazon. *Rev Inst Med Trop Sao Paulo* 2003; **45**(6): 327-31.

111. Rajgor DD, Gogtay NJ, Kadam VS, et al. Efficacy of a 14-day primaquine regimen in preventing relapses in patients with *Plasmodium vivax* malaria in Mumbai, India. *Trans R Soc Trop Med Hyg* 2003; **97**(4): 438-40.

112. Ruebush TK, 2nd, Zegarra J, Cairo J, et al. Chloroquine-resistant *Plasmodium vivax* malaria in Peru. *Am J Trop Med Hyg* 2003; **69**(5): 548-52.

113. Sumawinata IW, Bernadeta, Leksana B, et al. Very high risk of therapeutic failure with chloroquine for uncomplicated *Plasmodium falciparum* and *P. vivax* malaria in Indonesian Papua. *Am J Trop Med Hyg* 2003; **68**(4): 416-20.

114. Valibayov A, Abdullayev F, Mammadov S, et al. Clinical efficacy of chloroquine followed by primaquine for *Plasmodium vivax* treatment in Azerbaijan. *Acta Trop* 2003; **88**(1): 99-102.

115. Hapuarachchi HA, Dayanath MY, Abeysundara S, Bandara KB, Abeyewickreme W, de Silva NR. Chloroquine resistant falciparum malaria among security forces personnel in the Northern Province of Sri Lanka. *Ceylon Med J* 2004; **49**(2): 47-51.

116. Kurcer MA, Simsek Z, Zeyrek FY, et al. Efficacy of chloroquine in the treatment of *Plasmodium vivax* malaria in Turkey. *Ann Trop Med Parasitol* 2004; **98**(5): 447-51.

117. Leslie T, Rab MA, Ahmadzai H, et al. Compliance with 14-day primaquine therapy for radical cure of vivax malaria--a randomized placebo-controlled trial comparing unsupervised with supervised treatment. *Trans R Soc Trop Med Hyg* 2004; **98**(3): 168-73.

118. Vijaykadga S, Rojanawatsirivej C, Congpoung K, et al. Assessment of therapeutic efficacy of chloroquine for vivax malaria in Thailand. *Southeast Asian J Trop Med Public Health* 2004; **35**(3): 566-9.

119. Walsh DS, Wilairatana P, Tang DB, et al. Randomized trial of 3-dose regimens of tafenoquine (WR238605) versus low-dose primaquine for preventing *Plasmodium vivax* malaria relapse. *Clin Infect Dis* 2004; **39**(8): 1095-103.

120. Dunne MW, Singh N, Shukla M, et al. A double-blind, randomized study of azithromycin compared to chloroquine for the treatment of *Plasmodium vivax* malaria in India. *Am J Trop Med Hyg* 2005; **73**(6): 1108-11.

121. Genton B, Baea K, Lorry K, Ginny M, Wines B, Alpers MP. Parasitological and clinical efficacy of standard treatment regimens against *Plasmodium falciparum*, *P. vivax* and *P. malariae* in Papua New Guinea. *P N G Med J* 2005; **48**(3-4): 141-50.

122. Alvarez G, Pineros JG, Tobon A, et al. Efficacy of three chloroquine-primaquine regimens for treatment of *Plasmodium vivax* malaria in Colombia. *Am J Trop Med Hyg* 2006; **75**(4): 605-9.

123. Kurcer MA, Simsek Z, Kurcer Z. The decreasing efficacy of chloroquine in the treatment of *Plasmodium vivax* malaria, in Sanliurfa, south-eastern Turkey. *Ann Trop Med Parasitol* 2006; **100**(2): 109-13.

124. Maguire JD, Krisin, Marwoto H, Richie TL, Fryauff DJ, Baird JK. Mefloquine is highly efficacious against chloroquine-resistant *Plasmodium vivax* malaria and *Plasmodium falciparum* malaria in Papua, Indonesia. *Clin Infect Dis* 2006; **42**(8): 1067-72.

125. de Santana Filho FS, Arcanjo AR, Chehuan YM, et al. Chloroquine-resistant *Plasmodium vivax*, Brazilian Amazon. *Emerg Infect Dis* 2007; **13**(7): 1125-6.

126. Nateghpour M, Sayedzadeh SA, Edrissian GH, et al. Evaluation of Sensitivity of *Plasmodium vivax* to Chloroquine. *Iran J Public Health* 2007; **36**(3): 60-3.

127. Osorio L, Perez Ldel P, Gonzalez IJ. [Assessment of the efficacy of antimalarial drugs in Tarapaca, in the Colombian Amazon basin]. *Biomedica* 2007; **27**(1): 133-40.

128. Perez MA, Cortes LJ, Guerra AP, Knudson A, Usta C, Nicholls RS. [Efficacy of the amodiaquine+sulfadoxine-pyrimethamine combination and of chloroquine for the treatment of malaria in Cordoba, Colombia, 2006]. *Biomedica* 2008; **28**(1): 148-59.

129. Srivastava HC, Yadav RS, Joshi H, et al. Therapeutic responses of *Plasmodium vivax* and *P. falciparum* to chloroquine, in an area of western India where *P. vivax* predominates. *Ann Trop Med Parasitol* 2008; **102**(6): 471-80.

130. Teka H, Petros B, Yamuah L, et al. Chloroquine-resistant *Plasmodium vivax* malaria in Debre Zeit, Ethiopia. *Malar J* 2008; **7**: 220.

131. Carmona-Fonseca J, Maestre A. Prevention of *Plasmodium vivax* malaria recurrence: efficacy of the standard total dose of primaquine administered over 3 days. *Acta Trop* 2009; **112**(2): 188-92.

132. Liang GL, Sun XD, Wang J, Zhang ZX. [Sensitivity of *Plasmodium vivax* to chloroquine in Laza City, Myanmar]. *Zhongguo Ji Sheng Chong Xue Yu Ji Sheng Chong Bing Za Zhi* 2009; **27**(2): 175-6.

133. Nateghpour M, Edrissian G, Torabi A, et al. Monitoring of *Plasmodium vivax* and *Plasmodium falciparum* response to chloroquine in Bandar-Abbas district, Hormozgan province, Iran. [Arabic]. *Tehran Uni Med J* 2009; **67**(3): 178-83.

134. Orjuela-Sanchez P, da Silva NS, da Silva-Nunes M, Ferreira MU. Recurrent parasitemias and population dynamics of *Plasmodium vivax* polymorphisms in rural Amazonia. *Am J Trop Med Hyg* 2009; **81**(6): 961-8.

135. Rogers WO, Sem R, Tero T, et al. Failure of artesunate-mefloquine combination therapy for uncomplicated *Plasmodium falciparum* malaria in southern Cambodia. *Malar J* 2009; **8**: 10.

136. Carmona-Fonseca J. Vivax malaria in children: Recurrences with standard total dose of primaquine administered in 3 vs. 7 days *Iatreia* 2010; **23**(1): 10-20.

137. Dilmec F, Kurcer MA, Akkafa F, Simsek Z. Monitoring of failure of chloroquine treatment for *Plasmodium vivax* using polymerase chain reaction in Sanliurfa province, Turkey. *Parasitol Res* 2010; **106**(4): 783-8.

138. Takeuchi R, Lawpoolsri S, Imwong M, et al. Directly-observed therapy (DOT) for the radical 14-day primaquine treatment of *Plasmodium vivax* malaria on the Thai-Myanmar border. *Malar J* 2010; **9**: 308.

139. Yeshiwondim AK, Tekle AH, Dengela DO, Yohannes AM, Teklehaimanot A. Therapeutic efficacy of chloroquine and chloroquine plus primaquine for the treatment of *Plasmodium vivax* in Ethiopia. *Acta Trop* 2010; **113**(2): 105-13.

140. Congpuon K, Satimai W, Sujariyakul A, et al. *In vivo* sensitivity monitoring of chloroquine for the treatment of uncomplicated vivax malaria in four bordered provinces of Thailand during 2009-2010. *J Vector Borne Dis* 2011; **48**(4): 190-6.

141. Ketema T, Getahun K, Bacha K. Therapeutic efficacy of chloroquine for treatment of *Plasmodium vivax* malaria cases in Halaba district, South Ethiopia. *Parasit Vectors* 2011; **4**: 46.

142. Muhamad P, Ruengweerayut R, Chacharoenkul W, Rungsihirunrat K, Na-Bangchang K. Monitoring of clinical efficacy and *in vitro* sensitivity of *Plasmodium vivax* to chloroquine in area along Thai Myanmar border during 2009-2010. *Malar J* 2011; **10**: 44.

143. Van den Eede P, Soto-Calle VE, Delgado C, et al. *Plasmodium vivax* sub-patent infections after radical treatment are common in Peruvian patients: results of a 1-year prospective cohort study. *PLoS One* 2011; **6**(1): e16257.

144. Yohannes AM, Teklehaimanot A, Bergqvist Y, Ringwald P. Confirmed vivax resistance to chloroquine and effectiveness of artemether-lumefantrine for the treatment of vivax malaria in Ethiopia. *Am J Trop Med Hyg* 2011; **84**(1): 137-40.

145. Anez A, Navarro-Costa D, Yucra O, et al. [Therapeutic response of *Plasmodium vivax* to chloroquine in Bolivia]. *Biomedica* 2012; **32**(4): 527-35.

146. Graf PC, Durand S, Alvarez Antonio C, et al. Failure of Supervised Chloroquine and Primaquine Regimen for the Treatment of *Plasmodium vivax* in the Peruvian Amazon. *Malar Res Treat* 2012; **2012**: 936067.

147. Pedro RS, Guaraldo L, Campos DP, Costa AP, Daniel-Ribeiro CT, Brasil P. *Plasmodium vivax* malaria relapses at a travel medicine centre in Rio de Janeiro, a non-endemic area in Brazil. *Malar J* 2012; **11**: 245.

148. Leang R, Barrette A, Bouth DM, et al. Efficacy of dihydroartemisinin-piperaquine for treatment of uncomplicated *Plasmodium falciparum* and *Plasmodium vivax* in Cambodia, 2008 to 2010. *Antimicrob Agents Chemother* 2013; **57**(2): 818-26.

149. Manandhar S, Bhusal CL, Ghimire U, Singh SP, Karmacharya DB, Dixit SM. A study on relapse/re-infection rate of *Plasmodium vivax* malaria and identification of the predominant genotypes of *P. vivax* in two endemic districts of Nepal. *Malar J* 2013; **12**: 324.

150. Miahipour A, Keshavarz H, Heidari A, Raeisi A, Rezaeian M, Rezaei S. Assessment of the efficacy of 8 weeks of primaquine for the prevention of relapse in vivax malaria patients using SSCP-PCR and sequencing in South and South-East Iran, 2008-2011. *Trans R Soc Trop Med Hyg* 2013; **107**(7): 420-6.

151. Rios A, Alvarez G, Blair S. [Ten years of chloroquine efficacy for uncomplicated *Plasmodium vivax* malaria treatment, Turbo, Antioquia, 2002 and 2011]. *Biomedica* 2013; **33**(3): 429-38.

152. Zhu G, Lu F, Cao J, et al. Blood stage of *Plasmodium vivax* in central China is still susceptible to chloroquine plus primaquine combination therapy. *Am J Trop Med Hyg* 2013; **89**(1): 184-7.

153. Delgado-Ratto C, Soto-Calle VE, Van den Eede P, et al. Population structure and spatio-temporal transmission dynamics of *Plasmodium vivax* after radical cure treatment in a rural village of the Peruvian Amazon. *Malar J* 2014; **13**: 8.

154. Liu H, Yang HL, Tang LH, et al. Monitoring *Plasmodium vivax* chloroquine sensitivity along China-Myanmar border of Yunnan Province, China during 2008-2013. *Malar J* 2014; **13**: 364.

155. Ould Ahmedou Salem MS, Mohamed Lemine YO, Deida JM, et al. Efficacy of chloroquine for the treatment of *Plasmodium vivax* in the Saharan zone in Mauritania. *Malar J* 2015; **14**: 39.

156. Cheoymang A, Ruenweerayut R, Muhamad P, Rungsihirunrat K, Na-Bangchang K. Patients' adherence and clinical effectiveness of a 14-day course of primaquine when given with a 3-day chloroquine in patients with *Plasmodium vivax* at the Thai-Myanmar border. *Acta Trop* 2015; **152**: 151-6.

157. Yuan L, Wang Y, Parker DM, et al. Therapeutic responses of *Plasmodium vivax* malaria to chloroquine and primaquine treatment in northeastern Myanmar. *Antimicrob Agents Chemother* 2015; **59**(2): 1230-5.

158. Lo E, Nguyen J, Oo W, et al. Examining *Plasmodium falciparum* and *P. vivax* clearance subsequent to antimalarial drug treatment in the Myanmar-China border area based on quantitative real-time polymerase chain reaction. *BMC Infect Dis* 2016; **16**: 154.

159. Longley RJ, Sripoorote P, Chobson P, et al. High Efficacy of Primaquine Treatment for *Plasmodium vivax* in Western Thailand. *Am J Trop Med Hyg* 2016; **95**(5): 1086-9.

160. Mishra N, Srivastava B, Bharti RS, et al. Monitoring the efficacy of antimalarial medicines in India via sentinel sites: Outcomes and risk factors for treatment failure. *J Vector Borne Dis* 2016; **53**(2): 168-78.

161. Waqar T, Khushdil A, Haque K. Efficacy of Chloroquine as a first line agent in the treatment of uncomplicated malaria due to *Plasmodium vivax* in children and treatment practices in Pakistan: A Pilot study. *J Pak Med Assoc* 2016; **66**(1): 30-3.

162. Valecha N, Joshi H, Eapen A, et al. Therapeutic efficacy of chloroquine in *Plasmodium vivax* from areas with different epidemiological patterns in India and their *Pvdhfr* gene mutation pattern. *Trans R Soc Trop Med Hyg* 2006; **100**(9): 831-7.

163. Guthmann JP, Pittet A, Lesage A, et al. *Plasmodium vivax* resistance to chloroquine in Dawei, southern Myanmar. *Trop Med Int Health* 2008; **13**(1): 91-8.

164. Sutanto I, Suprijanto S, Nurhayati, Manoempil P, Baird JK. Resistance to chloroquine by *Plasmodium vivax* at Alor in the Lesser Sundas Archipelago in eastern Indonesia. *Am J Trop Med Hyg* 2009; **81**(2): 338-42.

165. Sutanto I, Endawati D, Ling LH, Laihad F, Setiabudy R, Baird JK. Evaluation of chloroquine therapy for vivax and falciparum malaria in southern Sumatra, western Indonesia. *Malar J* 2010; **9**: 52.

166. Heidari A, Keshavarz H, Shojaee S, Raeisi A, Dittrich S. *In vivo* Susceptibility of *Plasmodium vivax* to Chloroquine in Southeastern Iran. *Iran J Parasitol* 2012; **7**(2): 8-14.

167. Mishra N, Singh JP, Srivastava B, et al. Monitoring antimalarial drug resistance in India via sentinel sites: outcomes and risk factors for treatment failure, 2009-2010. *Bull World Health Organ* 2012; **90**(12): 895-904.

168. Gonzalez-Ceron L, Rodriguez MH, Sandoval MA, et al. Effectiveness of combined chloroquine and primaquine treatment in 14 days versus intermittent single dose regimen, in an open, non-randomized, clinical trial, to eliminate *Plasmodium vivax* in southern Mexico. *Malar J* 2015; **14**: 426.

169. Zuluaga-Idarraga L, Blair S, Akinyi Okoth S, et al. Prospective Study of *Plasmodium vivax* Malaria Recurrence after Radical Treatment with a Chloroquine-Primaquine Standard Regimen in Turbo, Colombia. *Antimicrob Agents Chemother* 2016; **60**(8): 4610-9.

170. Saravu K, Acharya V, Kumar K, Kumar R. *Plasmodium vivax* remains responsive to chloroquine with primaquine treatment regimen: a prospective cohort study from tertiary care teaching hospital in southern India. *Trop Doct* 2012; **42**(3): 163-4.

171. Valecha N, Savargaonkar D, Srivastava B, et al. Comparison of the safety and efficacy of fixed-dose combination of arterolane maleate and piperaquine phosphate with chloroquine in acute, uncomplicated *Plasmodium vivax* malaria: a phase III, multicentric, open-label study. *Malar J* 2016; **15**: 42.

172. Lee SW, Lee M, Lee DD, et al. Biological resistance of hydroxychloroquine for *Plasmodium vivax* malaria in the Republic of Korea. *Am J Trop Med Hyg* 2009; **81**(4): 600-4.

173. Krudsood S, Wilairatana P, Tangpukdee N, et al. Safety and tolerability of elubaquine (bulaquine, CDRI 80/53) for treatment of *Plasmodium vivax* malaria in Thailand. *Korean J Parasitol* 2006; **44**(3): 221-8.

174. Mohapatra MK, Padhiary KN, Mishra DP, Sethy G. Atypical manifestations of *Plasmodium vivax* malaria. *Indian J Malariol* 2002; **39**(1-2): 18-25.

175. Shalini S, Chaudhuri S, Sutton PL, et al. Chloroquine efficacy studies confirm drug susceptibility of *Plasmodium vivax* in Chennai, India. *Malar J* 2014; **13**: 129.

176. Carmona-Fonseca J, Uscategui RM, Correa AM. Vivax malaria in children: Clinical features and response to chloroquine. [Spanish]. *Colombia Medica* 2008; **39**(4): 364-77.

177. Daneshvar C, Davis TM, Cox-Singh J, et al. Clinical and parasitological response to oral chloroquine and primaquine in uncomplicated human *Plasmodium knowlesi* infections. *Malar J* 2010; **9**: 238.

178. Kolaczinski K, Durrani N, Rahim S, Rowland M. Sulfadoxine-pyrimethamine plus artesunate compared with chloroquine for the treatment of vivax malaria in areas co-endemic for *Plasmodium falciparum* and *P. vivax*: a randomised non-inferiority trial in eastern Afghanistan. *Trans R Soc Trop Med Hyg* 2007; **101**(11): 1081-7.

179. Barnadas C, Ratsimbasoa A, Tichit M, et al. *Plasmodium vivax* resistance to chloroquine in Madagascar: clinical efficacy and polymorphisms in *pvmdr1* and *pvcrt-o* genes. *Antimicrob Agents Chemother* 2008; **52**(12): 4233-40.

180. Pukrittayakamee S, Chantra A, Simpson JA, et al. Therapeutic responses to different antimalarial drugs in vivax malaria. *Antimicrob Agents Chemother* 2000; **44**(6): 1680-5.

181. Buchachart K, Krudsood S, Singhasivanon P, et al. Effect of primaquine standard dose (15 mg/day for 14 days) in the treatment of vivax malaria patients in Thailand. *Southeast Asian J Trop Med Public Health* 2001; **32**(4): 720-6.

182. Fryauff DJ, Leksana B, Masbar S, et al. The drug sensitivity and transmission dynamics of human malaria on Nias Island, North Sumatra, Indonesia. *Ann Trop Med Parasitol* 2002; **96**(5): 447-62.

183. Maguire JD, Lacy MD, Sururi, et al. Chloroquine or sulfadoxine-pyrimethamine for the treatment of uncomplicated, *Plasmodium falciparum* malaria during an epidemic in Central Java, Indonesia. *Ann Trop Med Parasitol* 2002; **96**(7): 655-68.

184. Tjitra E, Baker J, Suprianto S, Cheng Q, Anstey NM. Therapeutic efficacies of artesunate-sulfadoxine-pyrimethamine and chloroquine-sulfadoxine-pyrimethamine in vivax malaria pilot studies: relationship to *Plasmodium vivax dhfr* mutations. *Antimicrob Agents Chemother* 2002; **46**(12): 3947-53.

185. Tasanor O, Ruengweerayut R, Sirichaisinthop J, Congpuong K, Wernsdorfer WH, Na-Bangchang K. Clinical-parasitological response and *in-vitro* sensitivity of *Plasmodium vivax* to chloroquine and quinine on the western border of Thailand. *Trans R Soc Trop Med Hyg* 2006; **100**(5): 410-8.

186. Krudsood S, Tangpukdee N, Muangnoicharoen S, et al. Clinical efficacy of chloroquine versus artemether-lumefantrine for *Plasmodium vivax* treatment in Thailand. *Korean J Parasitol* 2007; **45**(2): 111-4.

187. Kinzer MH, Chand K, Basri H, et al. Active case detection, treatment of falciparum malaria with combined chloroquine and sulphadoxine/pyrimethamine and vivax malaria with chloroquine and molecular markers of anti-malarial resistance in the Republic of Vanuatu. *Malar J* 2010; **9**: 89.

188. Asih PB, Syafruddin D, Leake J, et al. Phenotyping clinical resistance to chloroquine in *Plasmodium vivax* in northeastern Papua, Indonesia. *Int J Parasitol Drugs Drug Resist* 2011; **1**(1): 28-32.

189. Maneeboonyang W, Lawpoolsri S, Puangsa-Art S, et al. Directly observed therapy with primaquine to reduce the recurrence rate of *Plasmodium vivax* infection along the Thai-Myanmar border. *Southeast Asian J Trop Med Public Health* 2011; **42**(1): 9-18.

190. Ganguly S, Saha P, Guha SK, et al. *In vivo* therapeutic efficacy of chloroquine alone or in combination with primaquine against vivax malaria in Kolkata, West Bengal, India, and polymorphism in *pvmdr1* and *pvcrt-o* genes. *Antimicrob Agents Chemother* 2013; **57**(3): 1246-51.

191. Liu H, Yang HL, Xu JW, Wang JZ, Nie RH, Li CF. Artemisinin-naphthoquine combination versus chloroquine-primaquine to treat vivax malaria: an open-label randomized and non-inferiority trial in Yunnan Province, China. *Malar J* 2013; **12**: 409.

192. Macareo L, Lwin KM, Cheah PY, Yuentrakul P, Miller RS, Nosten F. Triangular test design to evaluate tinidazole in the prevention of *Plasmodium vivax* relapse. *Malar J* 2013; **12**: 173.

193. Amaratunga C, Sreng S, Mao S, et al. Chloroquine remains effective for treating *Plasmodium vivax* malaria in Pursat province, Western Cambodia. *Antimicrob Agents Chemother* 2014; **58**(10): 6270-2.

194. Rajgor DD, Gogtay NJ, Kadam VS, et al. Antirelapse Efficacy of Various Primaquine Regimens for *Plasmodium vivax*. *Malar Res Treat* 2014; **2014**: 347018.

195. Assefa M, Eshetu T, Biruksew A. Therapeutic efficacy of chloroquine for the treatment of *Plasmodium vivax* malaria among outpatients at Hossana Health Care Centre, southern Ethiopia. *Malar J* 2015; **14**: 458.

196. Pareek A, Chandurkar N, Gogtay N, et al. Sustained Release Formulation of Primaquine for Prevention of Relapse of *Plasmodium vivax* Malaria: A Randomized, Double-Blind, Comparative, Multicentric Study. *Malar Res Treat* 2015; **2015**: 579864.

197. Beyene HB, Beyene MB, Ebstie YA, Desalegn Z. Efficacy of Chloroquine for the Treatment of Vivax malaria in Northwest Ethiopia. *PLoS One* 2016; **11**(8): e0161483.

198. Negreiros S, Farias S, Viana GM, et al. Efficacy of Chloroquine and Primaquine for the Treatment of Uncomplicated *Plasmodium vivax* Malaria in Cruzeiro do Sul, Brazil. *Am J Trop Med Hyg* 2016; **95**(5): 1061-8.

199. Seifu S, Zeynudin A, Zemene E, Suleman S, Biruksew A. Therapeutic efficacy of chloroquine for the treatment of *Plasmodium vivax* malaria among outpatients at Shawa Robit Health Care Centre, North-East Ethiopia. *Acta Trop* 2017; **171**: 44-51.
